# Oral Mucosal Epithelial Cells

**DOI:** 10.3389/fimmu.2019.00208

**Published:** 2019-02-14

**Authors:** Sabine Groeger, Joerg Meyle

**Affiliations:** Department of Periodontology, Justus-Liebig-University of Giessen, Giessen, Germany

**Keywords:** oral epithelial cells, differentiation, receptors, cytokines, immuno-modulation, infection, cancer

## Abstract

**Cellular Phenotype and Apoptosis:** The function of epithelial tissues is the protection of the organism from chemical, microbial, and physical challenges which is indispensable for viability. To fulfill this task, oral epithelial cells follow a strongly regulated scheme of differentiation that results in the formation of structural proteins that manage the integrity of epithelial tissues and operate as a barrier. Oral epithelial cells are connected by various transmembrane proteins with specialized structures and functions. Keratin filaments adhere to the plasma membrane by desmosomes building a three-dimensional matrix.

**Cell-Cell Contacts and Bacterial Influence:** It is known that pathogenic oral bacteria are able to affect the expression and configuration of cell-cell junctions. Human keratinocytes up-regulate immune-modulatory receptors upon stimulation with bacterial components. Periodontal pathogens including *P. gingivalis* are able to inhibit oral epithelial innate immune responses through various mechanisms and to escape from host immune reaction, which supports the persistence of periodontitis and furthermore is able to affect the epithelial barrier function by altering expression and distribution of cell-cell interactions including tight junctions (TJs) and adherens junctions (AJs).

In the pathogenesis of periodontitis a highly organized biofilm community shifts from symbiosis to dysbiosis which results in destructive local inflammatory reactions.

**Cellular Receptors:** Cell-surface located toll like receptors (TLRs) and cytoplasmatic nucleotide-binding oligomerization domain (NOD)-like receptors (NLRs) belong to the pattern recognition receptors (PRRs). PRRs recognize microbial parts that represent pathogen-associated molecular patterns (PAMPs).

A multimeric complex of proteins known as inflammasome, which is a subset of NLRs, assembles after activation and proceeds to pro-inflammatory cytokine release.

**Cytokine Production and Release:** Cytokines and bacterial products may lead to host cell mediated tissue destruction. Keratinocytes are able to produce diverse pro-inflammatory cytokines and chemokines, including interleukin (IL)-1, IL-6, IL-8 and tumor necrosis factor (TNF)-α. Infection by pathogenic bacteria such as *Porphyromonas gingivalis* (*P. gingivalis*) and *Aggregatibacter actinomycetemcomitans* (*A. actinomycetemcomitans*) can induce a differentiated production of these cytokines.

**Immuno-modulation, Bacterial Infection, and Cancer Cells:** There is a known association between bacterial infection and cancer. Bacterial components are able to up-regulate immune-modulatory receptors on cancer cells. Interactions of bacteria with tumor cells could support malignant transformation an environment with deficient immune regulation.

The aim of this review is to present a set of molecular mechanisms of oral epithelial cells and their reactions to a number of toxic influences.

## Introduction

The oral mucosal epithelium is a barrier that separates the underlying tissues from their environment. It consists of two layers, the surface stratified squamous epithelium and the deeper lamina propria. In keratinized oral mucosa, the epithelium is composed of the four layers stratum basale, stratum spinosum, stratum granulosum, and stratum corneum. In nonkeratinised epithelium, the stratum basale is followed by the stratum filamentosum and the stratum distendum. In the oral mucosa distinct phenotypes are differentiated, lining mucosa, masticatory mucosa, and specialized mucosa (1). Lining mucosa is localized over mobile structures such as soft palate, cheeks, lips, alveolar mucosa, vestibular fornix and floor of the mouth and is extensible and loosely bound to adjacent structures by an elastin rich connective tissue and has a non-keratinizing squamous epithelium. Masticatory mucosa is the rigid and tough protecting cover of the gingiva and the hard palate, tightly bound by dense connective tissue to the underlying bone. This epithelium is keratinized. Specialized mucosa is located on the dorsum of the tongue, shows a keratinized epithelium and includes lingual papillae and taste buds as specialized structures (2).

Junctional epithelium (JE) maintains the direct attachment to the tooth surface. The basal cells of the JE are attached to the connective tissue by the external basal lamina while the suprabasal cells are anchored to the tooth surface by an internal basal lamina that is produced by the JE. JE contains fewer cell-junctions as the oral gingival epithelium, but well developed gap junctions and some small adherens junctions can be detected (2). The JE has wide intercellular spaces, is highly permeable for water-soluble substances and serves as the primary pathway for the transmigration of polymorph nuclear leukocytes (3, 4). JE does not exhibit phenotypic stratification, but the outermost cells appear elongated and align with their long axis parallel to tooth surface (3).

The oral epithelial barrier is the outcome of numerous structural and functional protein interactions resulting in the ability to respond to a various exogenous, possibly toxic, influences. Squamous epithelia possess structural properties like stratification and cornification of the keratinocytes and specific cell-to-cell interactions to maintain its barrier function. It is now recognized that epithelial cells are not passive bystanders, but rather are metabolically active and capable of reacting to external stimuli by synthesizing a number of cytokines, adhesion molecules, growth factors, chemokines, and matrix-metalloproteases (5). Gingival tissues provide defense to resist frictional forces of mastication as well as to defend the soft tissues against chemical or microbial challenge (3).

## Cellular Phenotype and Apoptosis of Oral Epithelial Cells

**Figure 1:**
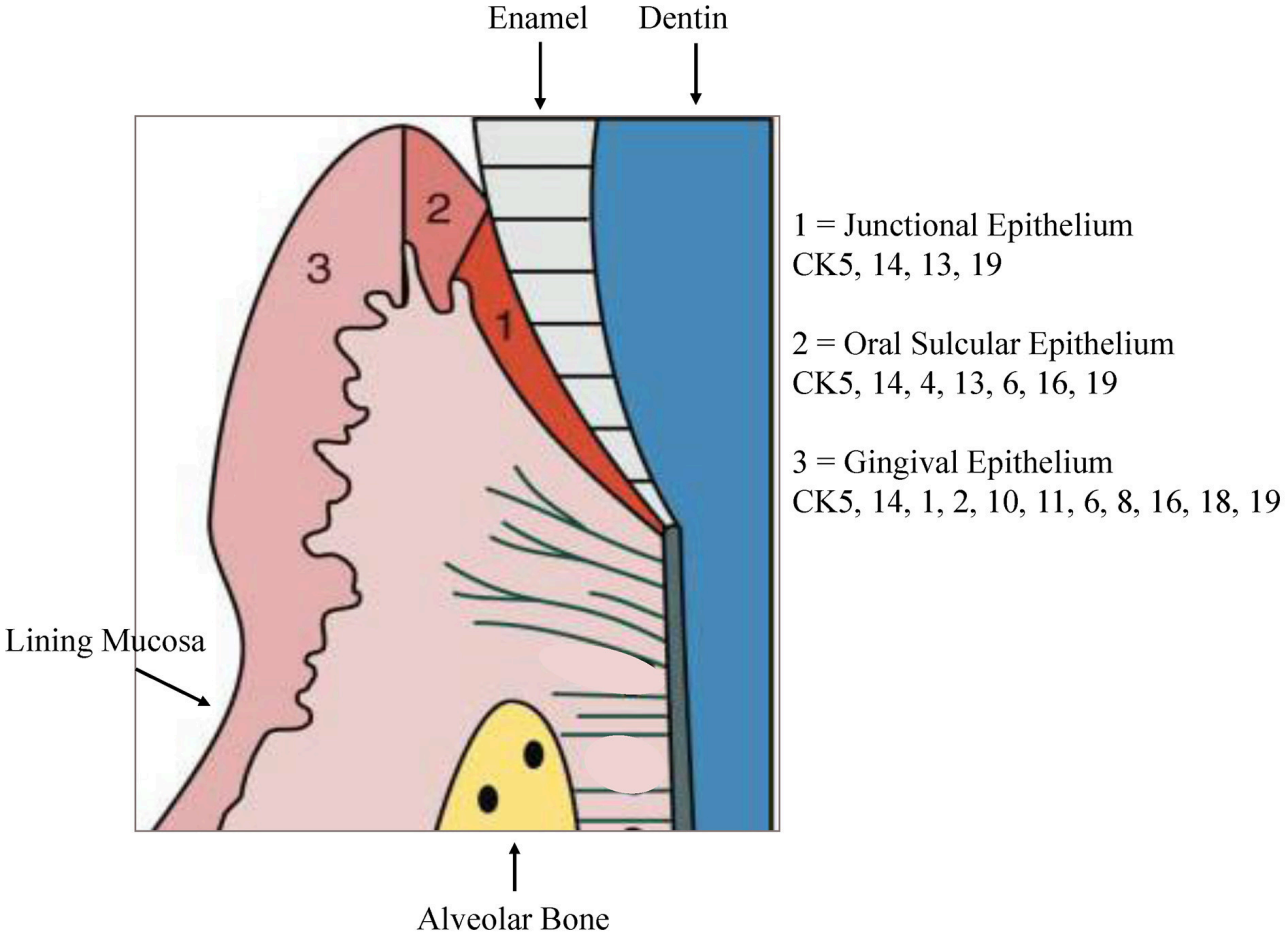
Cytokeratin distribution patterns. Cytokeratin (CK) distribution patterns in oral epithelia. Modified according to Pöllänen et al. (6).

The stratified epithelium of the oral mucosa belongs, together with the epithelium of the skin, to the most protective and resistant epithelia. It is composed of two layers, first epithelial cells with a basement membrane and second an underlying connective tissue, the *lamina propria* (4). The gingiva is combined of epithelial and connective tissues forming a collar of masticatory mucosa attached to the teeth and the alveolar bone. Gingival epithelium constitutes of a stratified squamous keratinized epithelium while the oral sulcular epithelium appears to be stratified and non-keratinized (Figure 1).

The non-keratinized JE shows no true phenotypic stratification (3). In contrast to the ortho-keratinized epidermis of the skin, oral epithelia normally express all three major differentiation patterns of keratinocytes. As an anatomical and functional unit, the gingival keratinization pattern shows variations that origin partly from adaptive processes of the tissue to the special site around fully erupted teeth. A keratinized epithelium similar to the epidermis is exhibited in regions that encounter masticatory and other mechanical forces. The muco-gingival junction designates the boundary of the gingiva from the movable alveolar mucosa and the mucosa of the floor of the mouth. The floor of the mouth and the buccal part need to be flexible for speech, swallowing or chewing and are covered with a lining mucosa that doesn't keratinize. The specialized mucosa on the dorsum of the tongue includes a number of papillae and is covered by an epithelium, which may be either keratinized or non-keratinized. Under physiological conditions, the barrier of polarized epithelia allows regulated paracellular fluxes of solutes and nutrients as well as the collection of antigens and surveillance by mucosal immune cells. During inflammation, this protective mechanism may be compromised by different stimuli originating from both sides of the epithelial barrier.

### Cytokeratins

Keratins are one major component of the epithelial cytoskeleton. They belong to the intermediate filament group of cytoskeletal proteins. A gene family of approximately 30 members encode keratins. They have a common structure composed of about 310-amino-acid central o-helical rod domain flanked by non-helical end-domains which are highly variable in sequence and structure (7). Based on the amino acid sequence and charge the keratin proteins are divided into two groups, acidic type I keratins including keratins K9-K20 and the basic or neutral type II keratins including K1–K8. Two keratin proteins, one type I and one type II, are always co-expressed and build heteropolymers to form the 10-nm keratin intermediate filaments (Ifs) that are part of the cytoskeleton. In the basal proliferative layer the keratin pair K5/K14 is expressed in stratified epithelia. Keratin 19 is detectable in simple epithelia and basal cells of non-keratinizing epithelia (8, 9). The keratin pair that is expressed in the post-mitotic layers of differentiating suprabasal cells differs depending on the localization. Cytokeratin distribution is highly specific and varies with type of epithelium, site, differentiation grade, so keratin expression is a sensitive and specific marker of differentiation in epithelial cells (10). Gingival and epidermal tissues as examples for cornifying epithelia, the keratins K1 and K10 are present while epithelia of buccal mucosa or esophagus K4 and K13 are the mainly expressed keratins (11). Suprabasal epithelial cells of the hard palate and gingiva furthermore express K2, designated as K2p in contrast to the epidermal K2e. The genes of K2p and K2e are related but separate (12). Other than the keratin pattern expressed from the attached gingiva some specialized epithelial cells within the gingiva show a distinct keratin pattern. The sulcular epithelium and cells of the gingival margin express K4 and K13. In contrast, the junctional epithelium adjacent to the tooth surface synthesizes K8, K13, K16, K18, and K19 (11, 13). Figure 1 shows the regional cytokeratin distribution pattern of the gingiva. Oral epithelia can exhibit one of 2 patterns of epithelial maturation, (1) they keratinize thus the mucosa matures by formation of a surface layer of keratin. This includes orthokeratinization which refers to the absence of nuclei in the superficial layer of scales on maturation and parakeratinization which designates the retention of pyknotic nuclei in the surface layer of squames during maturation (14). Nonkeratinization is the second possibility which means maturation with absence of a keratin layer which denotes that the nuclei remain, with scarce keratin filaments, in the cytoplasm of the most superficial cells (15). Depending on the functional demands, different types of keratinization are present in the gingival tissue. Oral gingival epithelium is keratinized while the sulcular and junctional epithelia (JE) as well as the lining mucosa are not keratinized (14).

Keratin filament assembly starts by parallel association. One type I chain form a paired dimer with its type II counterpart, two dimers associate in an antiparallel fashion to a tetramer. Two tetramers connect laterally resulting in a protofilament, from which eight are twisted into a rope building the keratin filament. Each keratin filament therefore possesses a cross section of 32 individual α helical coils. The polypeptide chains are further stabilized by strong lateral hydrophobic interactions. Bundled keratin filaments are associated to macromolecular networks that are oriented radial in the cytoplasm (2, 16). The regional specificity of keratin expression may be attributed to intrinsic specialization of regional keratinocyte stem cells. Disorders in keratin may be genetic or acquired. Numerous keratin mutations were identified as cause of several skin and mucosal disorders. Abnormal keratinization is part of several oral diseases. This topic is reviewed by Rao et al. (17).

### The Gingival Epithelial Barrier

Biofilms differentially modulate the epithelial cellular immune response based on their properties and composition. The bacterial biofilm located on the tooth surface and in the gingival crevice is considered as the primary causative agent involved in the pathogenesis of gingivitis and periodontitis and involves polymicrobial synergy and dysbiosis (18). Dysbiosis is based on the relative abundance of different bacterial species compared to their low presence in health, causing a modification of host–microbe interactions that can mediate destructive inflammation and bone loss (19, 20).

Keystone pathogens, such as *Porphyromonas gingivalis* (*P. gingivalis*), are able to subvert host response and promote breakdown of the homeostatic state, while further bacterial species show properties of pathobionts that can trigger destructive inflammation including both innate and adaptive immune response (21, 22). In addition the onset of gingivitis and periodontitis requires a susceptible host governing the complex inflammatory interactions.

**Figure 2:**
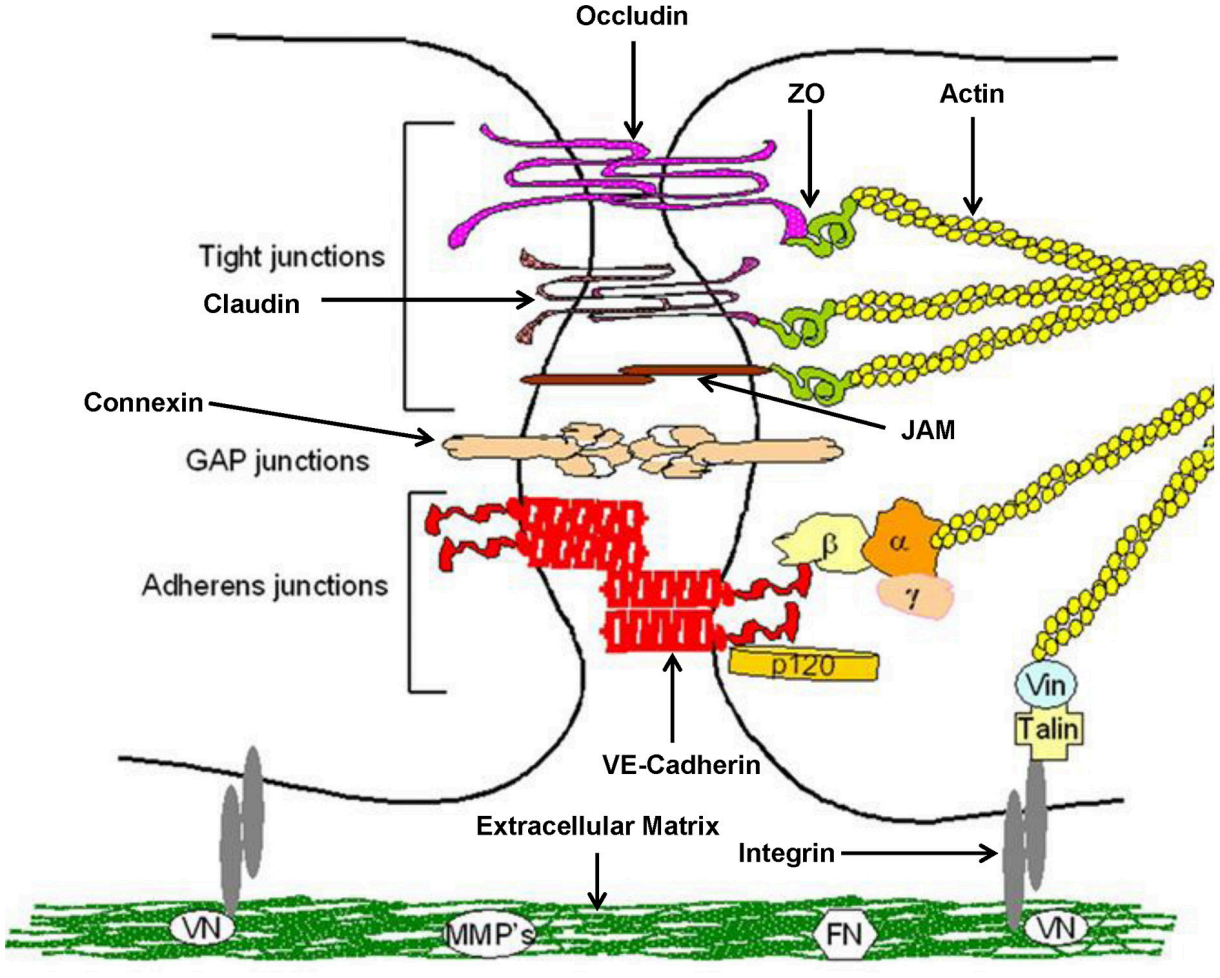
Structure of epithelial cell-cell contacts. Model of cellular junctions, modified according to Metha and Malik (24). Arrangement of junctions comprising tight junctions, adherens junctions, gap junctions, and integrin. Occludin, claudins, and junctional adhesion molecules (JAMs) required for tight junctions, whereas vascular endothelial (VE)-cadherin forms adherens junctions. Connexins are part of gap junctions. The extracellular domains of occludin, claudins, and VE-cadherin maintain cell-cell contacts. Intracellular domains provide junctional stability linking to the actin cytoskeleton via catenins (β,β-catenin, α,α-catenin; γ,γ-catenin; p120, p120-catenin) or zonula occludens-1 protein (ZO-1). Gap junctions are responsible for fast exchange of information by low-molecular-mass second messengers such as Ca^2+^ and IP_3_ between contiguous cells. Integrin receptors link endothelial cells with the extracellular matrix (ECM) through matrix proteins like fibronectin (FN) or vitronectin (VN). The cytosolic domains of integrins are linked to the actin cytoskeleton through the proteins talin and vinculin (Vin), involved in integrin-mediated signaling.

Keratinocytes of the gingival epithelium form a barrier against bacterial infection and invasion (23). They are interconnected by a number of specialized transmembrane molecular complexes, among them cell-cell junctions comprising tight junctions adherens junctions, and gap junctions (Figure 2).

Normal expression of these molecular complexes in the gingival tissues is essential for maintaining epithelial integrity. Once integrity is disturbed by biofilm-derived noxious influences, the associated bacteria may invade into the deeper periodontal tissues, triggering an inflammatory response. Thus these cell-cell connections are a crucial part of the innate immune response to resist microbial and toxic challenge.

### Structure and Function of Tight Junctions (TJ)

Tight junctions are complex protein structures, forming a belt like pattern among neighboring cells, encircling cells at the apical side of the lateral membrane (25). One of the major functions of the TJ complex is to form a barrier to regulate the passage through the paracellular pathway of water, ions, solutes and other small molecules (26–28). The structure of TJs appears as a string of continuous particles inlaid into the membrane, forming TJ strands. The strand is a fibril-like structure built by the assembly of claudin and tight junction–associated MARVEL proteins (TAMPs). TAMPS are composed of the tight junction-associated MAL = myelin and lymphocyte domain and the MARVEL = related proteins for vesicle trafficking and membrane link domain. The assembled structure represents the functional unit of TJs, formed by adjacent plasma membranes (29, 30).

A number of different signaling and trafficking molecules, that regulate cell differentiation, proliferation and polarity, are coordinated by TJs (31, 32). TJ topology consists of three protein domains, a helical transmembrane domain, a cytosolic scaffolding domain and cytosolic tail featuring cellular signaling. TJ strands are formed by the transmembrane proteins, a class that consists of multiple integral membrane proteins, including the groups of tissue- and cell-specific claudins, (29, 33) the TAMP family and the junction adhesion molecules (JAMs). The structure of claudin includes four transmembrane domains, two extracellular domains forming two loops, in which the N-terminus and C-terminus are located intracellularly. Claudins fulfill barrier properties (30, 34, 35) and are able to regulate the gate function as paracellular tight junction channels (PTJC). Their biological and physical properties are comparable to traditional ion channels (36). They also include occludin into the junctions (29). In adjacent mouse liver cells it was shown that various members of the claudin family form homophilic or heterophilic polymers. Furthermore, claudins may form paired strands to the membrane of adjacent cells (37). Differences in barrier properties between cell types are probably caused by different combinations of claudins (38).

The TAMP family includes MARVEL D1, also called occludin, MARVEL D2 (tricellulin) and MARVEL D3 protein. These molecules possess four transmembrane domains and two extracellular loops, similar to claudin (39, 40). It is not clear whether occludin composed strands have the same functions as strands formed by claudins but *in vitro* and *in vivo* studies demonstrated that occludin is of importance in TJ barrier function and intercellular adhesive interactions (39, 41–43). Claudin 1 and occludin were detected in the gingival but not in the sulcular and junctional epithelium. Furthermore, it was found that the adherens junction proteins P-cadherin and α-catenin are detectable in all three epithelia while E-cadherin was not present in junctional epithelium (44). The expression of claudin-4 was detected in the human oral squamous cell carcinoma epithelial cell line H413 (45) and in immortalized human gingival keratinocytes (46). Genetic investigations of adhesion proteins in stratified multi-layered gingival epithelial cell cultures showed strong expression of claudin-4, claudin-1, JAM-1, claudin-25, claudin-17, occludin and claudin-12 (47). Occludin is able to associate with different signaling molecules such as the non-receptor tyrosine kinase c-Yes, atypical protein kinase C (aPKC) and phosphoinositide 3-kinase (PI3K), as well as protein phosphatases 2A and 1 and appears to have signal transmitter functions (48, 49).

MARVEL D2 (also called tricellulin) is detectable at the tricellular contact sites. It is assembled to strands that form a tubular structure vertical to the bicellular TJ belt (50). It probably controls the flow of macromolecules but it is also needed for TJ organization. In the mouse mammalian epithelial cell line Eph4 tricellulin knock-out led to impairment of the structure of bicellular and tricellular contacts (51). The third TAMP, MARVEL D3, is expressed in many epithelial cells and its function was found not to be essential for TJ formation (52).

The members of the junctional adhesion molecule family (JAM), JAM-A, JAM-B, JAM-4, JAM-L and coxsackie and adenovirus receptor (CAR) belong to the immunoglobulin superfamily. JAMs seem to be less important for the regulation of the junctional structure, but rather contribute to adhesion and signaling. Studies mostly focused on the junctional role of JAM-A, that was shown to be localized to claudin-based tight junction fibrils in epithelial cells (53). JAM-A protein contains two extracellular immunoglobulin-like loops, a single transmembrane and a cytoplasmic domain ending in a PDZ binding motif that has been reported to interact with AF-6/afadin and zonula occludens protein (ZO)-1 (54) and ZO-2 (55). CAR appears to be a cell adhesion molecule that contributes to the formation of cell–cell contacts. In cultured epithelial cells, CAR molecules on adjacent cells form homotypic interactions (56). CAR is locally concentrated in TJs at the most apical regions of the lateral surfaces of polarized epithelial cells and its overexpression in cultured polarized cells increased TER (57), while soluble CAR and anti-CAR antibodies were shown to disrupt TJs, (56) suggesting that CAR is involved in the barrier function of TJs.

All members of the cytosolic scaffolding proteins have one or multiple post synaptic density proteins (PSD95), drosophila disc large tumor suppressor (Dlg1) domains, and zonula occludens-1 protein (PDZ) domains. They are able to bind to various integral membrane proteins like claudins, occludin or JAMs and also can bind to actin filaments. In this manner they connect TJ to the actin filaments and stabilize the protein complexes. An increasing number of PDZ containing proteins is known including membrane associated guanylate kinase (MAGUK)-like proteins, protein associated with Lin7 (Pals1), AF-6/afadin, atypical protein kinase C (aPKC), isotype-specific interacting protein (ASIP), partitioning-defective protein 3 (PAR-3), multi-PDZ protein 1 (MUPP1) and protein associated with tight junctions (PATJ) (58). These scaffolding proteins seem to be important for the organization and localization of TJs because blockade of PDZ domains results in poorly organized TJs that in consequence are distributed to other areas (59). Zonula occludens (ZO) proteins belong to the MAGUK family and include the three members ZO-1 (60), ZO-2 (61), and ZO-3 (62). Through their three PDZ domains ZO proteins interact with several proteins such as claudins, MARVEL D1 or JAMs and F-actin. This association with multiple proteins makes the formation of large complexes possible which are linking the cytoskeleton to the TJ strands (58).

The barrier function and structure of TJs is regulated by intracellular signaling proteins which include protein kinase A, protein kinase C, Rho kinase, myosin light chain kinase, GTPase Rab13, tyrosin kinase and mitogen activated protein kinase. All these proteins are not specific for TJs but essential for their establishment and function. The signal transduction of TJs is reviewed in Takano et al. (63). Further reviews are available addressing TJ physiology and function (64), regulation (65) and the specific components such as tricellular tight junctions (66). In gingival tissue, TJs were observed only in the granular and cornified layer, were they did not form complex strands, in contrast to cultured gingival keratinocytes *in vitro* that showed a largely extended framework of TJ strands (67).

**Figure 3:**
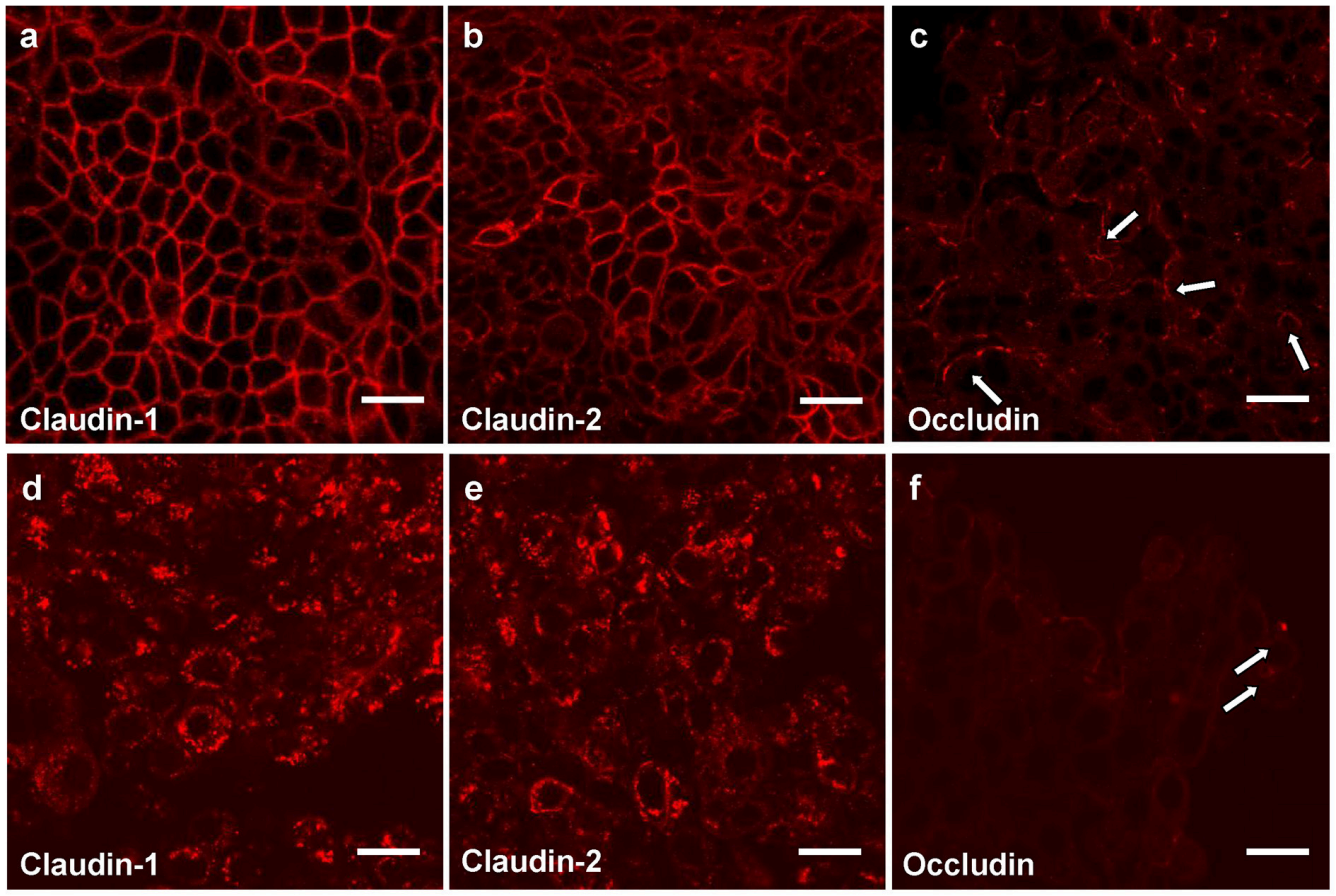
Tight junction proteins of primary keratinocytes after infection with *P. gingivalis*. Immunostaining of the tight junction proteins in primary human gingival keratinocytes, claudin 1 **(a,d)** claudin 2 **(b,e)** and occludin **(c,f)**; **(a–c)** cells in culture medium; **(d–f)** cells infected apically plus basolaterally with *Pophyromonas gingivalis* (*P. gingivalis*) W83 (MOI 10^4^) for 4 h. Arrows **(e,f)** show curved occludin strains in the walls of non-infected cells, in infected cells the arrows point to occludin aggregations, scale bar = 20 μm (71).

Measurement of the transepithelial electrical resistance (TER) is a method for investigations of the permeability of mucosal barriers *in vitro* and alterations of TER values are directly related to the integrity and function of the paracellular occluding barrier (68, 69). TER measurements are a useful tool to assess integrity of tight junctions. The strength of the transmucosal resistance is closely related to the number of junctional strands and junctional tightness (70). This correlation was demonstrated in primary human gingival keratinocytes (Figure 3) (67). The development of TER depends on the intracellular Ca^2+^ concentration (72).

Immortalized human gingival keratinocytes (IHGK) (73, 74) used in a 3D culture model, were infected with gingipain-producing *P. gingivalis* strains and a RGP/KGP defect mutant (75). This caused a significant decrease of TER after 24 h by the gingipain producing strains, but not by the defect mutant. Investigations of tight junction proteins in the same experimental setting using immunostaining revealed infection-induced alterations in claudin-1, claudin-2 and in occludin expressions. After infection the typical chicken wire pattern of claudin-1 and claudin-2 (Figures 3a,b) disappeared and the proteins formed conglomerates (Figures 3d,e). The curved strands of occludin present in the control assays (Figure 3c) were degraded as well (Figure 3f). Soluble virulence factors such as gingipains disrupt the epithelial barrier *in vitro*, which is correlated with the disintegration of junctional cell-cell complexes. Invasion and damage of the epithelial layer by infective agents is an important step and may result in bacterial invasion and destruction of the underlying connective tissue.

The results of this study give some insights into the initial stages of oral bacterial infections leading to gingivitis and periodontitis.

A further mechanism besides damage may be active internalization of epithelial adhesion complexes. In intestinal epithelial cells (T84 cells), IFN-γ induces a process of TJ protein internalization (claudin-1, occludin, JAM-A) by micropinocytosis, which results in leakage of the epithelial layer (76). Guo et al. (77) determined the impact of *P. gingivalis, P. gingivalis* LPS and eATP on TJ proteins in an oral epithelial cell culture model. Quantified real time polymerase chain reaction (RT-PCR), immunostaining and immunoblots of gene and protein expression in TJs revealed that *P. gingivalis* infection led to temporary upregulation of the genes encoding occludin, claudin- 1, and claudin-4 but not JAM-A, claudin-15, or ZO-1, while *P. gingivalis* LPS increased claudin-1, claudin-15, and ZO-1 and decreased occludin, JAM-A, and claudin-4. Significant upregulation of tight junction proteins was demonstrated when cells were pretreated with eATP. These results indicate that *P. gingivalis* induced early defense mechanisms of the host. *P. gingivalis* LPS stimulates the destruction of the epithelial barrier more potently than *P. gingivalis*. ATP stimulation further increased the effect on TJ proteins after *P. gingivalis* infection and LPS-induced disruption of epithelial integrity (77).

### Structure and Function of Adherens Junctions (AJ)

The adherens junctions (AJs) or zonula adherens, intermediate junction, or “belt desmosome,” are a defining feature of all epithelia, forming apical localized structures of adhesion closely aligned to the membranes of neighboring epithelial cells, that play an essential role in the regulation of the junctional complex. AJs are protein complexes that appear at cell–cell junctions in epithelial and endothelial tissues. Their localization is more basal than tight junctions. AJs are like bands that encircle the cell (zonula adherens) or appear as attachment spots to the extracellular matrix (adhesion plaques). The cell-to-cell adhesion sites are composed of cadherins which are connected to the actin cytoskeleton by catenins and other constituents like actinin and vinculin (78). AJs are formed by homophilic binding of the extracellular cadherin domains in a calcium-dependent manner. The cell-to cell apposition is maintained and reinforced by the homophilic interactions of single-pass transmembrane E-Cadherin (E-Cad) molecules. This process is stabilized by accumulating a tight network of actin filaments and by linking molecules that fix E-Cad clusters on the inner cytoskeleton (79). The E-Cad cytoplasmic domain consists of the β-catenin (β-Cat) interacting with p120-catenin (p120-Ctn). β-Cat associates with α-Catenin (α-Cad), maintaining the link to the actin cytoskeleton. Cadherin directly binds to β-catenin or plakoglobin, followed by binding to α-catenin that afterwards binds to vinculin, α-actinin, ZO-1 and actin (80). Also α-Cat is able to interact with further actin-binding proteins such as formin, AF6/afadin, or EPLIN (81). Nectin and its associated AF6/afadin protein L-afadin occurs concentrated at AJs (82). The transmembrane protein vezatin is localized at sites of cadherin-based cell-to-cell adhesion in cultured cells and anchors myosin VIIa to the cadherin-catenin complex (83). Due to their dynamic structure, adherens junctions physically connect adjacent epithelial cells, bridge intercellular adhesive contacts to the cytoskeleton, and are involved in the definition of each cell's apical–basal axis. E-Cads as well as catenins are substrates of phosphatases and kinases that are key regulators of AJs and modify the interactions between the proteins enabling them to regulate the interaction grade between E-Cad and the catenin complex and their concentrations in the membrane, which is essential for the modulation of adhesive strength and AJ remodeling [reviewed in (84)].

As a result of proteolytic disruption by putative periodontal pathogens such as *P. gingivalis* E-Cads are affected in periodontitis (85, 86). *P. gingivalis* is able to produce a variety of proteolytic enzymes, including eight endopeptidases and numerous exopeptidases (87). Gingipains are extracellular cysteine proteinases, which can impair endothelial cell adhesion (88, 89). Gingipains are also able to enhance collagenolysis by inducing matrix metalloproteinases of the host. Sheets et al. (90) also demonstrated cleavage of cellular receptors. The cleavage of adherence junction proteins (shown in HOK-16 cells) affects N-cadherin, VE-cadherin, β-integrin and reduces the adhesion of the cells to the extracellular matrix proteins (91). This may result in the detachment of the endothelial cells.

### Bacterial Adhesion and Invasion Into Epithelial Cells Are Pathological Processes Which Are Also Able to Disrupt the Epithelial Barrier in Periodontitis

It was shown that *P. gingivalis* fimbriae bind to cellular α5β1-integrin, which mediates bacterial adherence to host cells (92–94). Cellular integrins are heterodimeric receptors for extracellular matrix proteins and are essentially involved in cellular physiological processes that are related to metabolism, activation, differentiation, motility, and proliferation (95). These functions depend on the α5β1-integrin binding to its ligand fibronectin (96).

*P. gingivalis* can degrade cellular signaling molecules and inactivate a variety of cellular functions, which are important for healing and regeneration as well as homeostatic properties of periodontal tissues (91, 97–99).

Invasion of epithelial cells disrupts the epithelial barrier and the intracellular pathogens affect cellular functions by the usage of dynamin, actin fibers, microtubules, PI3K, and lipid rafts of the host cells.

Intracellular localization enables pathogens to penetrate deep into tissues by spreading from cell to cell, a process that seems to be mediated by membrane protrusions based on actin polymerization. This avoids the need of bacterial release into the extracellular space, i.e., periodontal pathogens like *P. gingivalis* spread in between cells without entering the extracellular space which may allow colonization of oral tissues avoiding revelation to the humoral immune response (86).

The importance of junctional proteins in the immune response to bacterial biofilms has been demonstrated by Belibasakis et al. (47). The group investigated the effects of a 10-species subgingival biofilm model on gene expression of all known cellular contacts (tight junctions, desmosomes, gap junctions and adherens junctions), and evaluated the involvement of the 3 “red-complex” species [*P. gingivalis, Treponema denticola* (*T. denticola*), and *Tannerella forsythia* (*T. forsythia*)] in a multi-layered gingival epithelial cell culture. The results of this study showed different effects on the junctional expression of the 2 biofilm (BF) models (one with and one without the “red complex”). It was found that BF including the “red complex” did not affect the expression of any of the studied tight junction genes. Absence of the “red complex” (RC) from the biofilm resulted in significantly higher Claudin-4 expression compared to the control after 3 and 24 h. Assessment of gene expression of desmosomes, adherens junctions, and gap junction proteins in response to biofilms without the “red complex” resulted in up-regulated desmocollin-2 expression after 3 h while the “red complex” including biofilm did not induce this effect. After 24 h, this expression was significantly downregulated by both biofilm variants. After 3 h of biofilm challenge, the gene expression of none of the investigated junctional adapter proteins was regulated while after 24 h, the expression of desmoplakin and plakoglobin were down-regulated in response to both biofilms (47).

## Cellular Receptors

### Toll-Like Receptors (TLRs)

**Figure 4:**
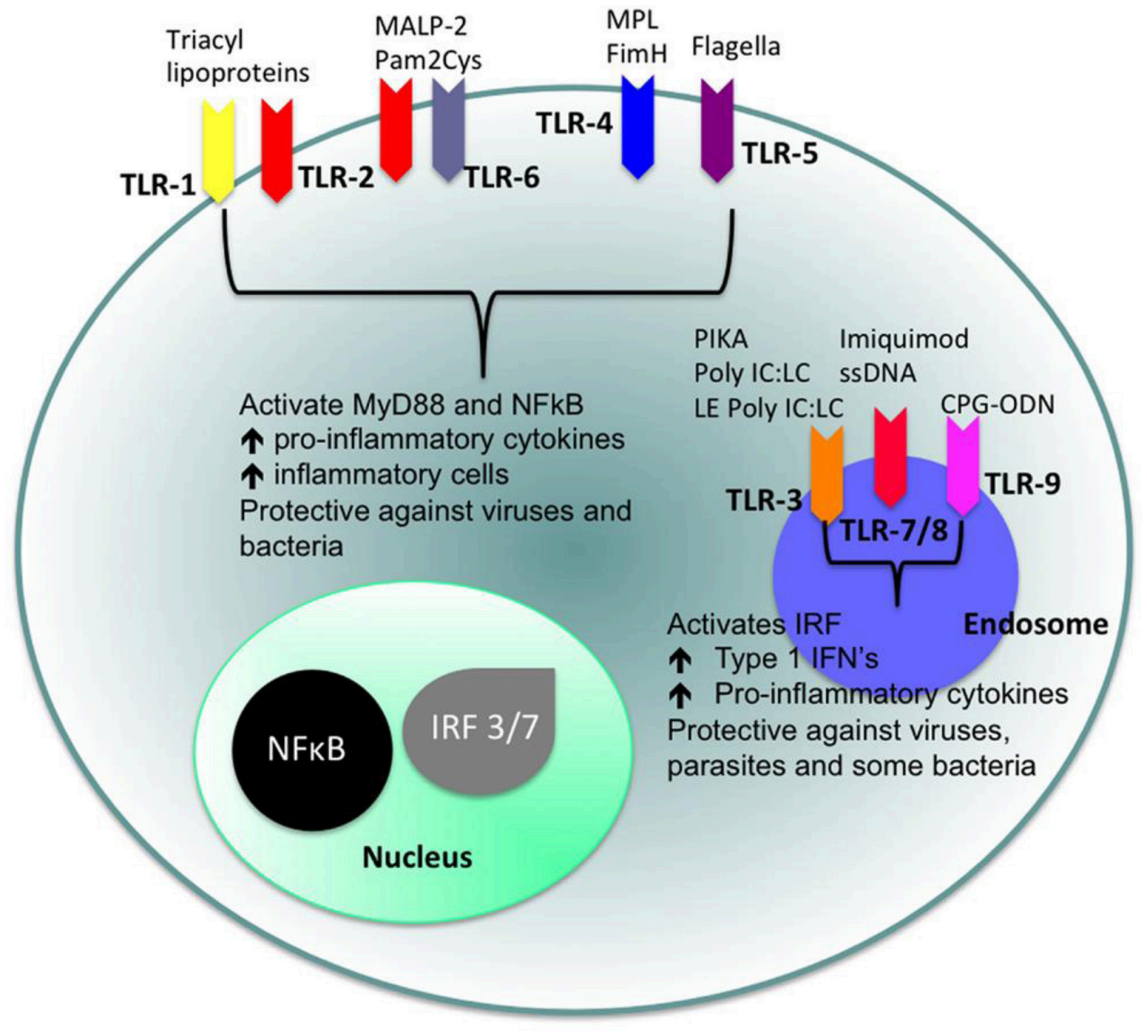
Cellular location of toll like receptors (TLRs) and the identity of their ligands/agonists. The stimulation of surface TLRs (TLR-2, TLR-4, and TLR-5) with appropriate ligands results in the activation of nuclear factor (NF)-κB. The ensuing increase in levels of pro-inflammatory cytokines and the influx of inflammatory cells then provides an environment, which protects against both virus and bacterial challenge. Activation of intracellular TLRs (TLR-3, TLR-7, TLR-8, and TLR-9) leads to interferon regulating factor (IRF) activation and the production of Type 1 interferons (IFNs) and pro-inflammatory cytokines, again providing an environment not conducive for pathogens (100).

**Figure 5:**
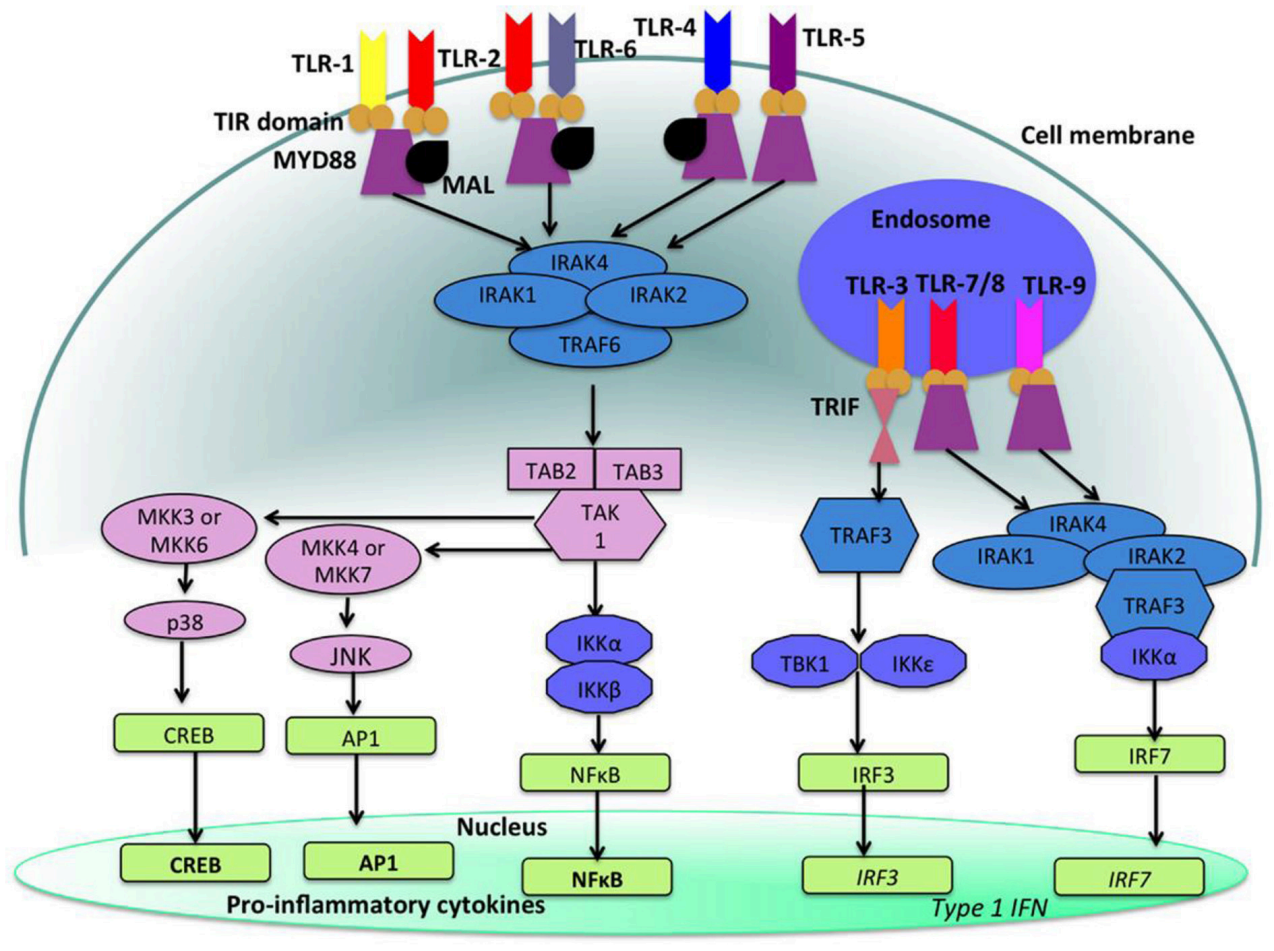
Toll like receptor (TLR)-signaling pathways. TLR-4, TLR-5, and the heterodimers TLR-1/TLR-2 and TLR-2/TLR-6 are located on the cell surface where they are activated by the appropriate ligand. Conversely, TLR-3, TLR-7, TLR-8, and TLR-9 are located within endosomal compartments of the cell and recognize microbial and viral nucleic acids. Stimulation of TLR-1/TLR-2, TLR-2/TLR-6, TLR-4, and TLR-5 leads to the engagement of myeloid differentiation primary response protein (MyD88) and MYD88-adapter-like protein (MAL) with the toll/interleukin-1 receptor (TIR) domain-containing adapter proteins. This stimulates downstream signaling pathways that involve the interactions between IL-1R-associated kinases (IRAKs) and the adapter molecules tumor necrosis factor (TNF) receptor-associated factors (TRAFs) and activates mitogen-activated protein kinases (MAPKs) JUN N-terminal kinases (JNK) and p38. Activation of these kinases leads to the activation of transcriptional factors such as nuclear factor-κB (NF-κB), cyclic adenosine mono phosphate (AMP)-responsive element binding protein (CREB), and activator protein-1 (AP-1). A major consequence of activation of surface TLRs is the induction of pro-inflammatory cytokines. Activation of TLR-7, TLR-8, and TLR-9 also leads to the engagement of MyD88, MAL, IRAKs, and NF-κB inhibitor kinase (IKK)α, however, interferon-regulatory factors (IRFs) are activated, which leads to the production of type 1 interferons (IFN). Stimulation of TLR-3 results in the association of TIR domain-containing adapter protein inducing IFNβ (TRIF). This leads to the downstream signaling of TNF receptor-associated factors (TRAFs) and IKK leading to the activation of IRF3 and the production of type 1 IFNs (100).

Toll-like receptors (TLRs) (Figures 4, 5) belong to the best characterized family of cellular effectors for the detection of pathogens (101). TLRs are widely expressed in eukaryotic cells. They are trans-membrane proteins that recognize molecular structures classified as “pathogen associated molecular patterns” (PAMPs) and thus belong to the pattern recognition receptors (PRRs). These patterns are present in nearly all types of microorganisms (102). Toll-like receptors contain a horseshoe-shaped extracellular leucine-rich repeat (LRR) and an intra-cytoplasmic toll/IL-1R (TIR) domain that are connected by a single trans-membrane domain. The LRR domain is responsible for ligand recognition and intracellular signal transfer is maintained by the TIR domain. TLRs represent not only the most important but also one of the first mechanisms in immune-defense against fungal, bacterial and viral pathogens. After binding the TLR downstream signaling pathway is activated playing an important role in innate and adaptive immune responses. In the oral cavity a great number of microorganisms is constantly present, therefore expression and function of TLRs is essential for the maintenance of oral tissue homeostasis. In humans, currently 10 TLRs have been identified, including extracellular as well as intracellular receptors. All exhibit a number of specific ligands, except for the orphan receptor TLR10, where the specific ligand has yet not been discovered (101, 103). TLR1, TLR2, TLR4, TLR5, TLR6, and TLR10 are expressed on the cell surface for recognition of extracellular microorganisms and ligands. TLR3, TLR7, TLR8, and TLR9 are intracellularly localized in the cytosolic endosomal compartment, binding microorganisms and ligands which passed the membrane of the host cell (104). Figure 4 shows the location of TLRs and the identity of their ligands/agonists. TLR11 has been identified in the human genome but does not translate into a protein, because its open reading frame contains a stop codon (105). TLR2 forms heterodimers with TLR1 or TLR6 and recognizes peptidoglycan, lipopeptide and lipoproteins while lipopolysaccharide of Gram-negative bacteria is the specific ligand of TLR4 (106, 107). TLR3 recognizes double-stranded RNA (dsRNA), TLR 5 can detect bacterial flagellin, TLR7 and TLR8 were shown to recognize imidazoquinilins and single-stranded RNA and TLR9 detects bacterial and viral DNA over their cytosine and guanine basepairing (108–113). LRR binding by ligands induces conformational changes of TIR resulting in interactions between TIR domains of adjacent TLRs and binding of additional adaptor proteins that are needed for the initiation of the intracellular signaling cascade. The most important adaptor molecules are the myeloid differentiation factor 88 (MyD88), the MyD88 adaptor-like (Mal) (TIR domain-containing adaptor protein, TIRAP), the TIR domain-containing adaptor protein inducing interferon-β (TRIF) (TIR-containing adaptor molecule, TICAM as synonym) and the TRIF-related adaptor molecule (TRAM) (114–119). TLR signaling can be negatively regulated by a variety of inhibitory molecules, including the toll-interacting protein (Tollip), interleukin-1 receptor (IL-1R) associated protein kinase (IRAK)-M, the sterile a- and HEAT-Armadillo- motif-containing protein (SARM), and the B cell adaptor or PI3K (BCAP), which inhibit downstream phases in the TLR- dependent signaling cascades. IL-1R associated protein kinases (IRAKs) IRAK4, IRAK1, and IRAK2 are activated by MyD88 followed by activation of tumor necrosis factor receptor-associated factor 6 (TRAF6) and RIP, that proceed by activation of transforming growth factor (TGF)-β-activated kinase 1 (TAK1) and TAK1-binding protein (TAB1, TAB2, and TAB3) complex (120–123). Subsequently, gene expression regulatory factors of the mitogen activated protein kinases (MAPK) family (ERK, JNK, p38) and NF-kB are activated, regulating cell survival and proliferation, and induce immune cell activation, production of pro-/anti-inflammatory mediators (cytokines and chemokines), interferons, and anti-microbial products. Activation of the intracellularly located TLR7, TLR8, and TLR9 is forwarded through MyD88 as well, but can also initiate TRAF6, IRAK4, and TRAF3-dependent activation of IRF7, which translocates to the nucleus and induces the production of type-I interferon (114, 124).

Figure 5 shows the signaling pathways of TLRs. mRNA of all 10 TLRs was detected in oral epithelial cells, but the actual expression and cellular localization of TLR proteins varies and is inducible. TLR2 is highly expressed in the basal layer of the gingival epithelium, levels are lower in the superficial layers that are more exposed to microorganisms and environmental influences. Apart from the detection of colonizing microorganisms in the superficial part of the epithelium, this may be regarded as a mechanism that facilitates TLR-depending inflammatory response only when pathogens are recognized in the basal layer. For TLR1, TLR3, TLR4, TLR5, and TLR9 a similar expression pattern was demonstrated (125, 126). The expression of TLR7 and TLR8 shows the same pattern in healthy and inflamed tissue. TLR2 and TLR4 expression is increased in acute and persistent gingival inflammation, though stimulation with TLR agonists did not induce production of pro-inflammatory cytokines, but β-defensin-2 generation in epithelial cells, and thus favored local downstream immune response (127).

Under chronic inflammatory conditions such as periodontitis in contrast to the TLR2 upregulation expression of TLR4 decreased, which may prevent from inflammatory exacerbation, i.e., tissue and bone destruction through containment of the inflammatory response (128). It was demonstrated that human healthy and inflamed oral tissues express TLR2, TLR4, NOD1 and NOD2 molecules, where cell-surface localizations of TLR2 and TLR4 could be more clearly detected in the inflamed than in healthy gingiva. It was furthermore demonstrated that human oral epithelial cell lines HSC-2, HO-1-u-1, and KB cells as well as primary cultured oral epithelial cells constitutively express TLR2, TLR4, NOD1, and NOD2. Stimulation of these cells with TLR and NOD agonists caused up-regulation of the antimicrobial peptide β-defensin (129). Oral epithelial cells, in contrast to colonic epithelial cells, did not secrete cytokines such as IL-8, monocyte chemoattractant protein-1 (MCP-1), granulocyte colony stimulating factor (G-CSF), granulocyte macrophage colony-stimulating factor (GM-CSF), and vascular endothelial growth factor (VEGF) after stimulation with bacterial components but upregulated expression of peptidoglycan recognition proteins (PGRPs), a further family of pattern recognition molecules (130, 131). These results suggest that part of the cells are desensitized to prevent tissue destruction over excessive innate immune responses to bacterial stimuli, because cells and bacteria interact constitutively (130, 131).

In periodontitis an abnormal immune response known as a “hyper-responsive” phenotype was demonstrated by investigations of peripheral blood leukocytes that were stimulated with TLR2 and TLR4 agonists. The stimulation resulted in elevated levels of pro-inflammatory cytokines produced by leukocytes that were derived from patients with localized aggressive periodontitis. This altered immune response may result in rapid loss of connective tissue and periodontal attachment as well as alveolar bone, which could result in early tooth loss already in young individuals (132). A cross-sectional study examined the role of epigenetic regulation, specifically DNA methylation status, of genes in the TLR pathway in patients with localized aggressive periodontitis (LAP). Peripheral blood stimulated with *Escherichia coli* (*E. coli*) LPS was analyzed for DNA methylation of seven TLR signaling genes. At specific CpG positions in LAP patients compared to healthy controls, differences in the methylation status were observed, as well as between severe and moderate LAP. Individuals with moderate LAP presented hypermethylation of both the upregulating and downregulating genes, while severe LAP presented hypomethylation of these genes. The methylation status correlated with an increased pro-inflammatory cytokine profile in LAP patients suggesting that epigenetic modifications in TLR signaling may modulate disease progression and tissue destruction (133).

A meta-analysis assessing the association between TLR4 polymorphisms and chronic periodontitis (CP) detected an association between TLR4C > G (rs7873784) allele and CP in Asians (134). The association between TLR4 polymorphisms and gastric cancer was investigated in the meta-analysis by Jin et al. (134). This group detected an increased gastric cancer risk in TLR4 + 896A/G and TLR4 + 1196C/T polymorphism in a Caucasian population (135).

### Nucleotide-Binding Oligomerization Domain Receptors (NODs)

**Figure 6:**
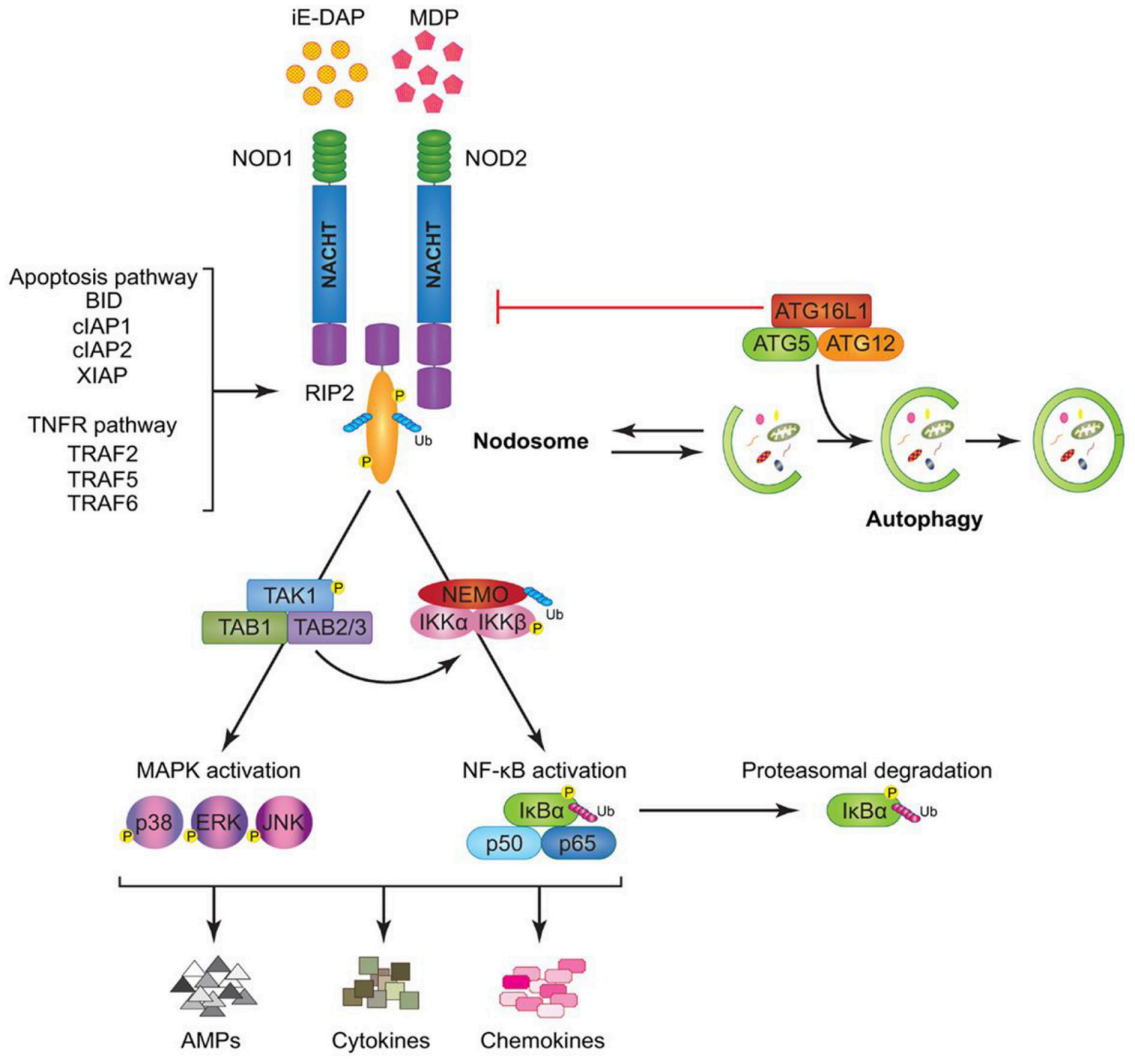
Model of Nucleotide-binding oligomerization domain-containing protein (NOD)1 and NOD2 signaling cascades. NOD1 and NOD2 recognize bacterial peptidoglycans (PGNs), (iE-DAP), and muramyl dipeptide (MDP), respectively. Following ligand sensing the NODs recruit their common adaptor receptor-interacting serine/threonine-protein kinase (RIP)2 by caspase activation and recruitment domains (CARD)–CARD interactions and induce RIP2 to undergo phosphorylation. The members of the tumor necrosis factor receptor-associated factor (TRAF) family (TRAF2, TRAF5, and TRAF6), the inhibitor of apoptosis (IAP) family (XIAP, cIAP1, and cIAP2), and the B-cell lymphoma (BCL)2 family (BID) bind to RIP2 and facilitate its ubiquitination allowing the recruitment of transforming growth factor-β-activated kinase (TAK)1 and ubiquitinated nuclear factor (NF-κB) essential modulator (NEMO) to the nodosome. On one hand, NEMO instigates activation of the canonical NF-κB pathway by phosphorylating NF-κB inhibitor kinase (IKK)α and IKKβ, by inducing nuclear factor of kappa light polypeptide gene enhancer in B-cells inhibitor alpha (IκBα) phosphorylation and proteasomal degradation, and by freeing p50 and p65 NF-κB subunits. On the other hand, transforming growth factor-β-activated kinase 1 (TAK1) recruits, transforming growth factor-β-activated kinase binding protein (TAB)1 and TAB2/3 inducing both (p38, extracellular-signal Regulated Kinases = ERK, and JUN N-terminal kinases = JNK) mitogen activated protein kinases (MAPK) and NF-κB activation. Stimulation of both arms culminates in the induction of anti-microbial peptides (AMPs), cytokines, and chemokines. The formation of the nodosome promotes autophagy and conversely, a fully functional autophagy machinery helps in signal transduction through the nodosome. Autophagy-related protein (ATG)16L1 along with ATG5 and ATG12 is required for autophagosome formation, however, independently of its autophagy functions, ATG16L1 negatively regulates NOD/RIP2 signaling (136).

Nucleotide-binding oligomerization domain receptors (NODs) (Figure 6) are cytosolic pattern recognition molecules that bind to peptidoglycan (PGN), a component of bacterial cell walls. They belong to the NOD-like receptor (NLRs) family including also NACHT-LRR (leucine-rich repeat) and pyrin-domain-containing proteins (NALPs), neuronal apoptosis inhibitor factors (NAIPs), and ICE-protease activating factor (IPAF) (137–139). The NOD1 ligand is PGN-derived γ-D-glutamylmesodiaminopimelicacid (iE-DAP) while muramyl dipeptide (MDP) is a NOD2 ligand (140, 141). MDP is detectable in Gram-negative and also in Gram-positive bacterial PGN, while iE-DAP is present in Gram-negative bacterial PGN and in PGN of particular Gram-positive bacteria such as *Bacillus subtilis* and *Listeria monocytogenes* (142). Hence, NOD1 is particularly involved in recognizing components from Gram-negative bacterial cell walls, while NOD2 can sense both (143, 144). A number of different cell types including oral epithelial cells express NOD1 that plays an essential role in innate immune responses (127, 129, 145). NOD1 binding and downstream signaling elicits an inflammatory reaction, inducing the production of cytokines, chemokines and antimicrobial peptides. Among these products, some are pro-inflammatory, such as interleukin (IL)-6, IL-8, tumor necrosis factor (TNF)-α and human beta defensin (hBD)-2, while others have immuno-regulatory or antimicrobial properties, such as interferon (IFN)-γ and human β-defensin-1 (hBD-1).The effects of iE-DAP on cytokine production have also been investigated with conflicting results: while it was reported that iE-DAP stimulated various human epithelial cells to produce anti-microbial peptides, but not pro-inflammatory cytokines like IL-6 and IL-8 (127, 129, 131, 145), it was also shown in human intestinal epithelial cells and dental pulp fibroblasts that NOD1 activation induces the production of pro-inflammatory cytokines (127, 142, 146–148).

In a human oral mucosal epithelial cell line (Leuk-1) upon stimulation activation of NOD1, receptor-interacting serine/threonine-protein kinase 2 (RIP2) and P-NF-κB was demonstrated, which was significantly inhibited by pretreatment of the cells with cigarette smoke extract (CSE). The suppressive effect of CSE on NOD1 expression was reversed following iE-DAP treatment. Combination of CSE stimulation with iE-DAP treatment prevented the further enhancement of RIP2 and P-NF-κB levels, i.e., iE-DAP reversed the inhibitory effect of CSE on NOD1 expression and prevented the over-activation of RIP2 and P-NF-κB due to CSE exposure. CSE furthermore upregulated levels of IL-6, IL-8, and TNF-α and downregulated IFN-γ level while iE-DAP enhanced the levels of IL-6, TNF-α, and IFN-γ, indicating that iE-DAP augmented gene expression and release of IL-6, TNF-α, and IFN-γ in Leuk-1 cells but diminished the mRNA level of IL-8 without affecting the production of IL-8 at protein level. These results indicate that iE-DAP is able to antagonize CSE-mediated effects on NOD1 expression and downstream signaling to a certain extent (149). In Figure 6 a model of NOD1 and NOD2 signaling cascades is depicted.

### Protease-Activated Receptors (PARs)

Protease-activated receptors (PARs) are a family of G-protein-coupled receptors (GPCRs) that include four members, PAR-1, PAR-2, PAR-3, and PAR-4, that play an important role in wound healing, inflammation, hemostasis, thrombosis, cancer progression, and embryonic development (150). PARs are activated by proteolytic cleavage of the N-terminal extracellular sequence of the receptors by a proteinase. This cleavage exposes a new N-terminal sequence, operating as a tethered ligand which, after binding to the receptor, initiates multiple signaling cascades (151–153). Although all PARs show the same mechanism of function, it has been demonstrated that different PARs can be activated by different proteinases and show diverse distributions and biological activities (154). One main activator of PAR-1, PAR-2, and PAR-3 is thrombin, further essential activators of PAR-1 comprise activated protein C (APC) and matrix metalloproteinase-1 (MMP-1). Trypsin and human mast cell tryptase activate PAR-2 while trypsin and cathepsin G activate PAR-4. Analysis of the downstream signaling responses after activation of PARs revealed that PAR-1, PAR-2, and PAR-4 may signal autonomously, while PAR-3 rather seem to be a co-receptor for PAR-1 and PAR-4 (155–158). PARs are expressed in a number of different cell types and it has been suggested that they influence physiological processes, such as growth, development, inflammation, tissue repair, and pain. In gingival epithelial cells (GEC) the presence of PAR-1,-2 and 3 mRNA and protein expression could be demonstrated while PAR-4 was not detected. Pre-incubation of the cells with *P. gingivalis* supernatant containing proteolytic activity, induced PAR-2 mRNA up-regulation. In contrast, PAR-1 and -3 were down-regulated. The authors concluded from these results that GECs recognize *P. gingivalis* by PARs and mediate innate immunity cell responses (159). PARs, NODs, and TLR were found to exhibit a complex interplay. In silencing experiments it was shown that knock-out of one receptor type may affect the others. GEC with silenced PAR-1 and -2 reacted with up-regulated NOD1 and NOD2 expression upon stimulation with *P. gingivalis* or *Fusobacterium nucleatum* (*F. nucleatum*). Expression of TLR2 decreased after infection with *P. gingivalis* when PAR2 was knocked down but was not affected after stimulation with *F. nucleatum*, while TLR4 expression was increased after PAR2 silencing and subsequent stimulation with *F. nucleatum*. *F. nucleatum* activates TLR4, while *P. gingivalis*, due to its unique LPS structure, is able to utilize TLR2 and shut down TLR4. These data suggest that if PAR receptors are absent, expression of TLRs is modified in response to bacteria following their activation level. PRRs can function as substitute in epithelial immune-response to bacterial challenge. These responses show variations depending on the properties of the bacterial stimuli (160).

### Inflammasomes

**Figure 7:**
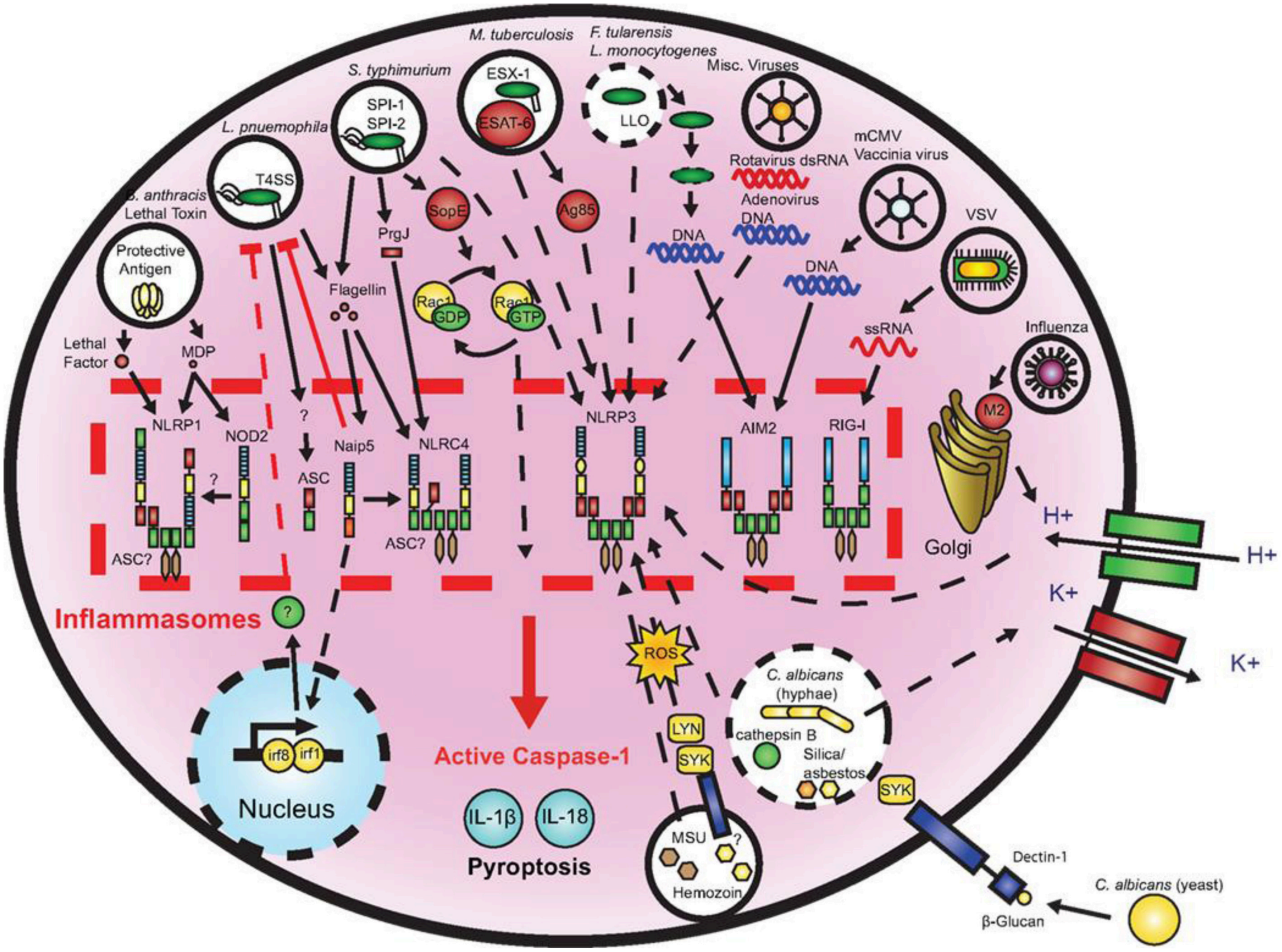
Microbial activation of the inflammasomes. Pathogenic microorganisms activate the inflammasomes through multiple agonists and pathways. *Salmonells typhimurium (S. typhimurium*)*, Legionells pneumophila (L. pneumophila*), and *Mycobacterium tuberculosis* (*M. tuberculosis*) reside within the host cell phagosome and are capable of activating inflammasomes through secreted flagellin, effectors, or undefined NACHT, LRR, and PYD domains-containing protein (NLRP)3 agonists. NACHT = NAIP, neuronal apoptosis inhibitor protein; C2TA, class 2 transcription activator, of the MHC; HET-E, heterokaryon incompatibility; TP1, telomerase-associated protein 1; LRR, leucine-rich repeat; PYD, PYRIN domain. *Francisella tularensis* (*F. tularensis*) and *Listeria monocytogenes* (*L. monocytogenes)*, which escape the phagosome activate absent in melanoma (AIM)2 that senses cytosolic deoxyribonucleic acid (DNA). *Bacillus anthracis* (*B. anthracis*) lethal toxin activates the NLRP1 inflammasome. *Candida. albicans* (*C. albicans*) and hemozoin activate NLRP3 through Spleen tyrosine kinase (SYK) signaling. Viral-mediated inflammasome activation is heavily dependent on the detection of nucleic acids by NLRP3, AIM2, and retinoic acid-inducible gene (RIG)-I. Dotted lines indicate signaling through an unknown mechanism (161).

**Figure 8:**
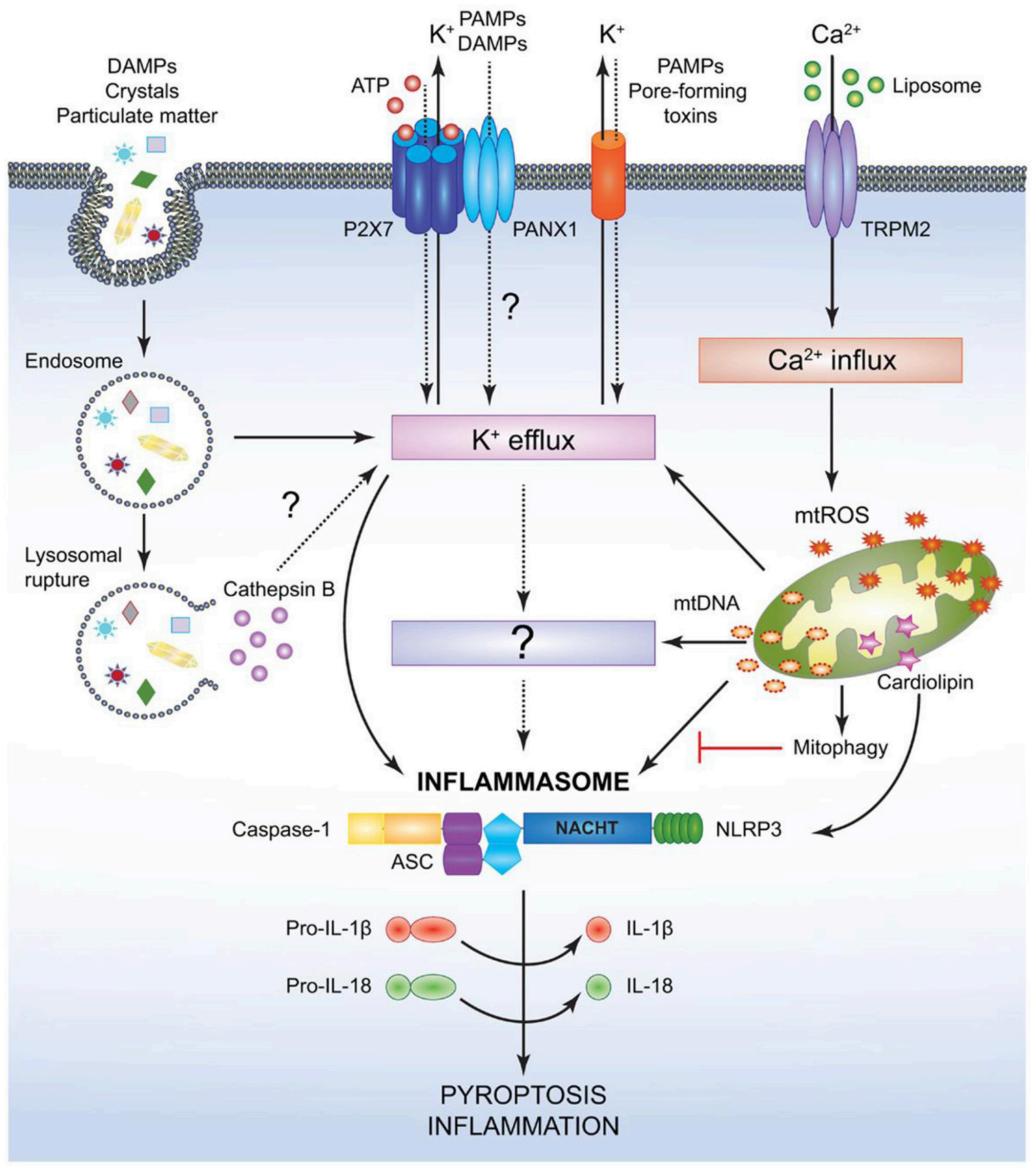
Mechanism for canonical NACHT, LRR and PYD domains-containing protein (NLRP)3- inflammasome activation. NACHT = NAIP, neuronal apoptosis inhibitor protein; C2TA, class 2 transcription activator, of the MHC; HET-E, heterokaryon incompatibility; TP1, telomerase-associated protein 1; LRR, leucine-rich repeat; PYD, PYRIN domain. Various pathogen associated molecular patterns (PAMPs) and damage associated molecular patterns (DAMPs) provide the signal 2 required to assemble and activate the NLRP3 inflammasome comprised of NLRP3, apoptosis-associated speck-like protein (ASC), and caspase-1. Although the precise mechanism leading to NLRP3 activation is still controversial, it is speculated that potassium ion K^+^ efflux may be the common cellular response that triggers inflammasome activation. However, this notion has not been fully verified and it is possible that an unidentified or intermediate adaptor may be required for transmitting signals between K^+^ efflux and the NLRP3 inflammasome. Crystals and particulate DAMPs enter the cell via endocytosis directly inducing K^+^ efflux and NLRP3-inflammasome formation. In addition, the endo- lysosomes carrying these DAMPs undergo lysosomal rupture and release cathepsin B, which acts as an intracellular DAMP and can induce K^+^ efflux. However, contradicting studies indicate that lysosomal rupture may cause K^+^ efflux and inflammasome activation even in the absence of cathepsin B. Adenosin triphosphate (ATP) binds to the P2X purinoceptor 7 (P2X7) receptor on the cell membrane and causes opening of the annexin 1 (PANX1) channels allowing K^+^ efflux and influx of any PAMPs and DAMPs present in the extracellular space. PAMPs such as pore-forming toxins activate the NLRP3 inflammasome and facilitate K^+^ efflux. Liposomes instigate Ca^2+^ influx through opening of (TRPM2) channels. Accumulation of excessive Ca^2+^ in the cytosol causes mitochondrial dysfunction and release of mitochondrial reactive oxygen species (mtROS) and oxidized mitochondrial deoxyribonucleic acid (mtDNA), which may activate the NLRP3 inflammasome either directly or by inducing K^+^ efflux. Clearance of distressed mitochondria by mitophagy serves to evade such inflammasome activation. Mitochondrial cardiolipin binds to NLRP3 and is required for the NLRP3-inflammasome activation. Following NLRP3-inflammasome assembly, caspase-1 undergoes proximity driven proteolytic cleavage and further processes pro-interleukin (IL)-18 and pro-IL-1β into their mature active forms. Activation of the NLRP3-caspase-1 axis results in inflammation and pyroptotic cell death (136).

One further mechanism of the immune system to initialize a pro-inflammatory response is the so called inflammasome (Figures 7, 8), a protein complex composed as multimer which develops in the cytoplasm participating in the immune response to pathogenic microbes or danger signals. The inflammasome induces the production and secretion of mature pro-inflammatory cytokines, IL-1β and IL-18 eventually leading to pyroptosis, a special kind of cell death (162–165). Inflammasomes can be grouped into canonical and noncanonical pathways (162, 164–167). Typically, a functional canonical inflammasome complex consists of a nucleotide-binding domain leucine-rich repeat (NLR) protein, an adaptor molecule apoptosis-associated speck-like protein containing a CARD (ASC) domain, and caspase-1 (167). The composition of the upstream regulators and specific molecules of the inflammasome depends on the type of the danger signals and the microbial inducers (164). The best characterized NLR, the pyrin domain containing 3 (NLRP3) inflammasome (Figure 8) for example is activated by extracellular adenosine triphosphate (eATP) danger signaling by the purinic receptor 2X7 (P2X7) and reactive oxygen species (ROS). Infection with bacterial pathogens can induce the NLRP3 inflammasome. Specific activation of the absent in melanoma (AIM)2 inflammasome is triggered by sensing double-stranded DNA in the cytosol while the IL-1β-converting enzyme (ICE) protease-activating factor (IPAF) inflammasome is activated by Gram-negative bacteria (e.g., *Salmonella typhimurium, Shigella flexneri, Legionella pneumophila*, and *Pseudomonas aeruginosa*) which have type III or IV secretion systems (164). In gingival epithelial cells *F. nucleatum* also activates the NLRP3 inflammasome, which in turn activates caspase-1 and stimulates secretion of mature IL-1β (168).

*P. gingivalis* is able to inhibit the innate immune response using a nucleoside-diphosphate kinase (NDK) after stimulation with extracellular (e)ATP. The danger signal eATP binds to P2X7 receptors leading to activation of the inflammasome and caspase-1. Thus, exposure of gingival epithelial cells (GECs) to wild-type *P. gingivalis* was demonstrated to result in blockade of ATP-induced caspase-1 activation while NDK-deficient *P. gingivalis* showed fewer effects.

*P. gingivalis* NDK was shown to modify release of high-mobility group protein B1 (HMGB1), another pro-inflammatory danger signal, which, in non-infected cells, remains linked to chromatin. Infection with wild-type or NDK-deficient *P. gingivalis* induced release of HMGB1 from the nucleus to the cytosol. HMGB1 was delivered to the extracellular space when non-infected GECs were stimulated with ATP. HMGB1 was released in higher extend, when ATP-treated cells were infected with NDK-deficient mutants instead of wild-type *P. gingivalis*. These results suggest that NDKs are significantly involved in inhibiting P2X7-dependent inflammasome activation and HMGB1 release from bacterially infected GECs (169).

A multitude of inflammasomes exists, which can be activated by varying mechanisms resulting in the maturation and secretion of pro-inflammatory cytokines (170, 171). Extracellular ATP, one of the first activators that was discovered to induce NLRP3 inflammasome formation, is considered to belong to the group of endogenous damage-associated molecular patterns (DAMPs) released by dying or injured cells (172, 173). It exhibits minor presence in healthy tissues, but may increase to high micromolar concentrations at inflamed sites following tissue damage (174). Studies demonstrated that eATP caused caspase-1 activation that was followed by IL-1β release (175, 176). Yilmaz et al. (177) revealed that LPS-treated or infected gingival epithelial cells (GECs) did not secrete IL-1β unless they were stimulated with eATP and that eATP did not alter NLRP3 or apoptosis-associated speck like protein (ASC) expression in *P. gingivalis* infected gingival epithelial cells. NLRP10 is the smallest human NLR protein. It is different from the other NLR proteins because of its lack of the leucine-rich repeat domain, which is involved in ligand sensing or binding. Upon infection with two periodontal pathogens, *T. forsythia* and *F. nucleatum* the human oral epithelial cell line HOK-16B reacted with up-regulated mRNA and protein expression of NLRP10 while infection with *Streptococcus oralis* (*S. oralis*) did not induce this effect. These results demonstrate that NLRP10 up-regulation in HOK-16B cells is pathogen-specific (178). Figure 7 demonstrates microbial activation of the inflammasomes.

## Cytokine Production and Release

Interleukin (IL)-8 response of gingival epithelial cells after exposure to different multispecies biofilms was differentially regulated (179). Characterization of the whole secretome after biofilm challenge with species of the red complex demonstrated that more proteins were downregulated than up-regulated (180).

Keratinocytes are able to produce a variety of cytokines such as IL-1, IL-6, IL-8, and tumor necrosis factor (TNF)-α. They maintain normal homeostatic mechanisms and can induce proliferative effects upon injury. Mucosal cytokines may have pro-inflammatory as well as anti-inflammatory functions. An imbalance in the cytokine levels can support inflammatory diseases. Cytokines provide a paracrine (between adjacent cells), an endocrine (cells at distant sites) and an autocrine (intercellular) cell-to-cell communication system. Cytokines, based on different functions, origins, and chemical structures, are classified into the following groups: ILs, TNFs, chemokines, colony-stimulating factors (CSF), interferons (IFNs), and growth factors (GF). Cytokines share a multitude of activities and functions (pleiotropic and redundant), thus they could be classified in more than one group. IL-3 for example may also be classified in the CSF group (181). The IL-1 cytokines (IL-1α, IL-1β, and IL-1Ra) are important in regulation of immune response and inflammation because they induce the expression of many effector proteins, e.g., cytokines/chemokines, nitric oxide synthetase, and MMPs (182).

The immortalized human oral epithelial cell line OKF6-TERT2 responds to co-incubation with *in vitro* cultured biofilms of single- and mixed-bacterial species consisting of *P. gingivalis, F. nucleatum, Aggregatibacter actinomycetemcomitans* (*A. actinomycetemcomitans*) and *Streptococcus mitis* (*S. mitis*) with increased expression of mRNA for IL-8, C-X-C motif chemokine ligand 3 (CXCL3), CXCL1, IL-1, IL-6, CSF-2, and TNF-α. The response was biggest after stimulation with mixed-species biofilms (183).

The chemokine IL-8 shows crucial importance in oral health because it supports the transition of activated immune cells into and through gingival tissues, and promotes immune cell adhesion, tissue remodeling, and angiogenesis (184). IL-8 is increased in the saliva of patients with oral carcinomas. It could probably be a biomarker for the detection of oral and oropharyngeal squamous cell carcinomas (185). In patients with severe periodontitis, IL-8 was also detected in high levels in crevicular fluid at healthy sites (186). Schueller et al. investigated the basal release levels of IL-8, and linked it to the bacterial community, personal oral hygiene and nutrition in persons with a healthy gingival situation (187). It was shown that the basal IL-8 release was between 9.9 and 98.2 pg/ml, and bacterial biofilms were distinctive for healthy oral microbiota. An association between basal IL-8 levels and the oral microbiota was detected, suggesting a link between oral bacteria and the inflammatory state. A link between nutrition, personal oral hygiene, oral microbiota and IL-8 levels was also reported. The identification of indicator bacteria in healthy subjects with high levels of IL-8 release was regarded as important as they possibly are promising indicators for the onset of oral diseases (187). Fujimura et al. (188), reported that the hemophoric hemoglobin receptor (HbR), that binds hemoglobin and captures porphyrin and heme (*P. gingivalis* needs iron to grow), in interaction with host cells, affected cellular signal transduction of these cells, followed by inhibited differentiation of osteoclasts from bone marrow macrophages (188). The IL-8 inducing function of HbR from host epithelial cells was demonstrated to be maintained by activation of cellular signal transduction. Increased expression of IL-8 by gingival epithelial cells was induced by HbR in a dose dependent manner. This process is associated with activation of p38 MAPK and Erk1/2 using silencing (si)RNAs and inhibitors (189).

IL-33 belongs to the IL-1 cytokine family and is constitutively expressed in the nuclei of epithelial and endothelial cells (190). Epithelial cell-derived IL-33 augments T helper cell (Th)2 cytokine-mediated inflammatory immune response upon bacterial challenge (191). IL-33 was detected in inflamed gingival epithelium from chronic periodontitis patients. Enhanced IL-33 expression induced by *P*. *gingivalis* was detected in the cytoplasm of human gingival epithelial cells *in vitro*. In contrast, *P*. *gingivalis* fimbriae, lipopolysaccharide or lipopeptide did not induce this effect. Inhibition of *P*. *gingivalis* proteases (gingipains) blocked IL-33 mRNA induction. Also the *P*. *gingivalis* gingipain-null mutant KDP136 did not up-regulate IL-33 expression. Silencing of PAR-2 and inhibition of phospholipase C, p38 and NF-κB restrained the *P*. *gingivalis* induced IL-33 expression. These results indicate activation of the PAR-2/IL-33 axis in human gingival epithelial cells by *P*. *gingivalis* via a gingipain-dependent mechanism (192).

The angiopoietin-like protein (ANGPTL), belongs to a family of eight secreted glycoproteins, but doesn't bind to the tyrosine kinase with immunoglobulin-like and EGF-like domains (Tie)2 angiopoietin receptor or to the related protein Tie1, and, classified as orphan ligand, appears to exhibit biological functions different from angiopoietins (193–195). ANGPTL2 manages tissue homeostasis by induction of inflammation and angiogenesis (194, 196). Elevated ANGPTL2 concentrations are present in gingival crevicular fluid (GCF) from chronic periodontitis patients and stimulation with *P. gingivalis* LPS up-regulated ANGPTL2 mRNA and protein levels in gingival squamous cell carcinoma Ca9-22 cells. Recombinant human ANGPTL2 caused augmented IL-1β, IL-8, TNF-α mRNA and protein levels in Ca9-22 cells. Silencing of ANGPTL2 and blocking antibodies against the ANGPTL receptor integrin α5β1 inhibited the IL-1β, IL-8, and TNF-α mRNA and protein up-regulation, which suggests that ANGPTL secretion induces inflammatory cytokines in gingival epithelial cells through an autocrine loop. Thus, a new inflammatory cytokine induction cascade featuring sequential *P*. *gingivalis* LPS- ANGPTL2-integrin α5β1 activation was detected which might be responsible for periodontal destructive processes induced by gingival epithelial cells. Thus ANGPTL2 participates in the pathogenesis of periodontitis and may promote continuous chronic inflammation (197).

An immortalized human gingival cell line reacted with enhanced IL-8 and IL-6 mRNA concentrations and supported phosphorylation of ERK and p38 MAP kinase upon infection with *A. actinomycetemcomitans* (198).

*A. actinomycetemcomitans*, a member of the taxonomic family *Pasteurellaceae*, has been related to the development of aggressive periodontitis and may also promote chronic periodontitis (199–201). Amongst other pathogenic members of the periodontal biofilm, *A. actinomycetemcomitans* produces various substances that are able to damage cells and tissues in a direct or indirect manner. As a member of the oral biofilm this bacterium is known to express complex operons for two cytotoxins, leukotoxin (Lkt) and cytolethal distending toxin (Cdt) (202, 203). These toxins are able to impair the host's immune response and thus may promote the pathogenesis of periodontitis (204). Human gingival epithelial cells (HGECs) were stimulated with 50 clinical strains and 7 reference strains of *A. actinomycetemcomitans*, including various serotypes and non-serotypeable strains, strains from deep or shallow pockets, and reference serotype strains, and investigated for the expression of IL-1β, IL-6, IL-8, and TNF-α mRNAs. Results showed that IL-8 mRNA was strongly up-regulated after stimulation with clinically obtained *A. actinomycetemcomitans* and also with reference strains. Serotype f induced the highest expression in comparison to the other serotypes. The JP2-like leukotoxin promoter gene and non-serotypeable (NS) 1 and NS2 caused lesser IL-8 induction compared to serotypeable strains, and IL-8 up-regulation after stimulation with clinical strains from deep pockets showed also significantly lower levels than those isolated from shallow pockets. These results indicate that JP2-like leukotoxin NS1 and NS2 from clinical isolates of *A. actinomycetemcomitans*, obtained from deep pockets, are able to affect neutrophil function by lowering the IL-8 responses, which results in immunosuppression that may support virulence and survival of these bacteria (205).

### Influence of *Treponema denticola*

*T. denticola* is a Gram-negative anaerobic oral spirochete that is known as a member of periodontal pathogens and is associated with chronic periodontitis (206, 207). It possesses a variety of virulence factors including dentilisin, an active cell-surface-located protease that cleaves at phenylalanyl/alanyl and prolyl/alanyl bonds, trypsin-like protease activity and the capability for motility and chemotaxis via periplasmic flagella [for Review see Dashper et al. (208)].

On primary gingival epithelial cells it has been demonstrated that *T. denticola* fails to induce IL-8 production that can't be explained by IL-8 degradation, as a protease mutant that does not degrade IL-8 also didn't induce IL-8 production. *T. denticola* furthermore failed to promote transcription of IL-8 and hβD-2 mRNA. This impaired epithelial cell response to *T. denticola* suggests contribution to the pathogenesis of periodontitis by deficient chemotaxis initiation of neutrophils into the periodontal pocket (209). The mechanism of IL-8 suppression by *T. denticola* was investigated using immortalized human gingival epithelial (HOK-16B) cells. Dentisilin degraded TNF-α, an IL-8-inducing cytokine, suggesting modulation of IL-8 (210). In monocytes derived from human peripheral blood mononuclear cells the role of *T. denticola* periplasmic flagella (PF) was investigated. Stimulation of the innate immune response via PAMPs revealed, that flagella-exhibiting wild type *T. denticola* induced the production of the cytokines TNF-α, IL-1, IL-6, IL-10, and IL-12 over activation of nuclear factor (NF)-κB through toll like receptor (TLR)2. These results suggest that *T. denticola* activates the innate immune response in a TLR2-dependent way and that flagella are involved as key bacterial components (211).

The IL-17 family, consisting of IL-17A–IL-17F, plays an important role in host defense against microbial challenge and has also been demonstrated to be crucial in pathogenesis of periodontitis (212). Initially IL-17A was regarded as a cytokine exclusively expressed by Th17 cells (213) but subsequent studies revealed that other cellular sources are capable to express IL-17A, including γδ T cells, natural killer cells, neutrophils, eosinophils, mast cells and macrophages (212). In gingival tissues of periodontitis patients (214–217) presence of IL-17 producing cells correlates with severity of inflammation in periodontitis lesions (218). Furthermore, elevated IL-17A levels were detected in GCF of patients with periodontitis. The IL-17A levels are reduced after non-surgical therapy (219, 220). Awang et al. (221) analyzed clinical linkage between cytokines of the IL-17 family and periodontitis and the biological effect of IL-17A and IL-17E using *in vitro* model systems. According to their studies serum, saliva and GCF IL-17A levels are increased in periodontitis patients and correlate with the clinical parameters attachment loss, pocket depth and bleeding on probing. Periodontitis patients exhibit lower IL-17E serum levels and the IL-17A-IL-17E ratio in serum also correlates positively with clinical parameters. *In vitro*, IL-17E suppressed IL-17A and *P. gingivalis* induced chemokine-expression by inhibiting phosphorylation of the NF-kB p65 subunit, which indicated that in the pathogenesis of periodontitis the serum IL-17A-IL-17E ratio might be a marker of disease severity while IL-17E is opposing IL-17A. IL-17E produced by oral keratinocytes may down-regulate IL-17A in the periodontium (221).

## Immuno-modulation, Bacterial Infection and Cancer Cells

### Role of Growth Arrest-Specific 6 (GAS6)

The growth arrest-specific 6 (GAS6) and Protein S (PROS1) are ligands of the tyrosin-protein kinase receptor (TYRO)3, AXL, and proto-oncogene (MERTK or TAM) receptor tyrosine kinases (222), which are involved in a number of biological processes including immune regulation (223). GAS6 is constitutively expressed in oral epithelial cells and was shown to downregulate epithelial activation at equilibrium state in order to sustain homeostasis (224). In the oral mucosa, the superficial layers of the epithelium express GAS6 together with its predominant receptor AXL. After birth GAS6 expression is induced in a MYD88-dependent way by the developing microbiota. GAS6 expressed by dendritic cells (DCs) was shown to inhibit IL-6 production by supporting development of T regulatory (Treg) cells and diminishing Th17 cell generation. This provides a more tolerogenic immunological environment for the oral microbiota (224). GAS6/AXL signaling seems to play a crucial role in the regulation of homeostasis in the oral mucosa. Thus, pathogens affecting the GAS6/AXL axis might generate a dysbiotic state and subsequent oral pathology. The induction of oral adaptive immune responses by specific pathogens is abolished in *Gas6*–/– mice and GAS6 is able to induce simultaneously pro- and anti-inflammatory regulatory pathways after mice were infected with *P. gingivalis*. GAS6 not only upregulates the expression of adhesion molecules in blood vessels which supports extravasation of immune cells belonging to the innate immune response, it also increases the expression of CCL19 and CCL21 chemokines and thus supports oral DCs to migrate to the lymph nodes. In addition the expression of the pro-inflammatory molecules P-selectin, intercellular adhesion molecule 1 (ICAM-1), and vascular cell adhesion molecule 1 (VCAM-1) in the oral mucosa is downregulated by GAS6. Furthermore, GAS6 blocks DC maturation and decreases antigen presentation by DCs to T cells. The authors concluded that GAS6 facilitates migration of inflammatory cells and DCs through the endothelium in both directions, while T-cell stimulation and activation of the mucosa is inhibited. This highly regulated activity of GAS6 supports a rapid but still moderate mucosal immunity to oral pathogens (225).

### Bacterial Infection and Cancer Cells

Evidence suggests an increased risk for cancer in chronic infections and inflammation. Bacterial infections and carcinogenesis seem to be connected (226). Periodontitis, one of the most common chronic human inflammatory diseases, is caused by microorganisms in the oral biofilm that trigger local inflammation. Periodontitis induces epithelial proliferation and apical migration along the root surface of the tooth and leads to a constant release of inflammatory cytokines, growth factors, prostaglandins, and enzymes, which all of them are closely associated with the development of cancer (226). In previous studies by Tezal et al. (226, 227) it was reported that the assumed association between periodontitis and oral neoplasms is significant. High PD-L1 expression has been linked to different types of human malignancies like lung cancer, pancreatic cancer, oral cancer, kidney cancer, breast and gastric cancer (228, 229). Head and neck cancer belongs to the 10 most frequent cancers worldwide (230). About 95% of the cases are of the squamous cell carcinoma (SCC) type. Tumors can only grow if their tissue environment provides them with a milieu that sustains their growth and spread. Alterations of tissue homeostasis by infection or inflammation can compromise stromal structural integrity and support tumorigenesis (231).

Programmed death ligand 1 (PD-L1, also called B7-H1) belongs to the B7 family and plays an important role in the regulation of cell-mediated immune response (232, 233). PD-L1 mediated signals are essential in co-signaling of T cell activation and tolerance (234). PD-L1 signals are also able to downregulate T cell functions and survival (228, 235). Modification of immune responses in cancer sites is a crucial mechanism that is linked to immune evasion of tumors. In the tumor microenvironment PD-L1 and programmed death receptor 1 (PD-1) may interact and induce tumor-protective mechanisms by activation of multiple specific pathways including ligation of PD-1 by PD-L1 on antigen specific T cells. This in turn may lead to functional anergy and/or apoptosis of these effector T cells. Ligation of PD-1 by PD-L1 possibly promotes tolerance and directly protects the tumor from apoptosis by reverse signaling through PD-L1 (228, 236, 237). It was demonstrated that *P*. *gingivalis* W83 up-regulates PD-L1 in oral cancer cells and in primary as well as in immortalized human gingival keratinocytes (238). High levels of PD-L1 were demonstrated in invasive oral squamous carcinoma cells (239). Also, positive PD-L1 expression was detected in tissue samples of oral squamous cell carcinomas *ex vivo* (240). Membrane proteins of *P*. *gingivalis* are responsible for the up-regulation while cytosolic proteins failed to induce PD-L1 (241). Primary human gingival keratinocytes (PHGK) and oral squamous cell carcinoma (SCC-25) cells up-regulated a number of inflammation-related genes upon infection with *P. gingivalis* membranes, amongst them members of the downstream NF-κB signaling pathway, TLR signaling and MAPK pathways. These data not only suggest that *P. gingivalis* membrane induces a pro-inflammatory response in malignant and non-malignant oral epithelial cells, but also indicates a possible link between infection and oral carcinomas, since p38 MAPK and MEK4-JNK1 signaling pathways were shown to be involved in context of tumor microenvironment and the control of cancer growth (242).

In a Chinese population an increased risk for head and neck carcinomas in individuals with oral submucous fibrosis, oral leukoplakia and repetitive dental ulcers was demonstrated suggesting a strong association between these diseases and cancer (243). Also patients with colorectal cancer carry strains of *F. nucleatum* in the cancerous lesions. *F. nucleatum* is one of the most densely colonized bacterial species in the oral cavity and known to be associated with periodontitis (244). The effect of oral pathogens on the development of oral tumors was investigated by Hoppe et al. (245). Stimulation of oral tumor cells with *P. gingivalis* led to increased cell proliferation while in contrast, *A. actinomycetemcomitans* promoted cell death. Bacteria as well as anti-microbial peptides induced diverse effects on the transcription levels of the oncogenic defensin genes and epidermal growth factor receptor (EGFR) signaling. The primary impact of the two oral pathogens were opposite on the proliferation behavior of oral tumor cells. In contrast, both induced similar secondary effects on the proliferation rate by modulating the extend of oncogenic important α-defensin gene expression. Human defensins differently modify epidermal growth factor receptor signaling, supporting the assumption that these anti-microbial peptides are possible ligands of EGFR. Interaction of these two molecules may cause the modification of the proliferative behavior of oral tumor cells (245). A newly established model of long term infection by stimulating human immortalized oral epithelial cells (HIOECs) with *P. gingivalis* at a low multiplicity of infection (MOI) for 5–23 weeks was used to investigate possible alterations in tumors. Persistent infection with *P. gingivalis* induced changes in cell morphology, enhanced proliferative capability and promoted cell migratory and invasive properties. Furthermore, tumor-related genes including GAS6 and PD-L1 that possibly act as key regulators in transformation from non-cancerous to tumor cells were upregulated as a reaction to long-term exposure of *P. gingivalis*. The authors concluded that *P. gingivalis* is able to support tumorigenic characteristics of HIOECs, suggesting that chronic *P. gingivalis* infection possibly represents a risk factor for oral cancers (246). Using a newly-established murine oral tumorigenesis model that is associated with periodontitis, it was reported that chronic bacterial infection supports development of OSCC, inducing enhanced signaling of the IL-6- signal transducer and activator of transcription 3 (STAT3) pathway. The results indicate that periodontal pathogens like *P. gingivalis* and *F. nucleatum* promote tumorigenesis by interacting directly with oral epithelial cells through TLR2. Furthermore, these pathogens stimulate the proliferation of human OSCC and induce expression of molecular key factors that take part in tumorigenesis, such as STAT3, that becomes activated in response to interferons, EGF, IL-5, IL-6, and cyclin D1, which is required for progression through the G1 phase of the cell cycle and induces cell migration. These findings represent a function of oral bacteria in the mechanism of chemically induced OSCC tumorigenesis (247).

The epithelial–mesenchymal transition (EMT) is critical in the conversion of normal epithelial cells into carcinoma cells during carcinogenesis. Downregulation of E-cadherin and up-regulation of N-cadherin and the transcription factors zinc finger protein SNAI1 (= Snail), SNAI2 (= Slug) and zinc finger e-box-binding homeobox (Zeb)1 are typical markers for this conversion (248). In a newly established long term infection *in vitro* model Lee et al. (249) demonstrated that primary oral epithelial cells after 120 h of infection with *P. gingivalis* develop an EMT phenotype including decrease of E-cadherin and increase of Slug, Snail, and Zeb1 expression. Infection of primary rat epithelial cell cultures with periodontal pathogens for 8 days caused an enhanced percentage of vimentin-positive cells, 20% after stimulation with *P. gingivalis* and 30% after infection with *F. nucleatum*. Furthermore, the periodontal pathogens induced augmented activation of Snail and the electrical impedance in comparison with unexposed controls of the cultures was reduced. The capability of the cells to migrate was extended as reaction to bacterial stimulation, demonstrated by the number of migrating cells and scratch-wound closure rates. In conclusion, persistent stimulation of primary rat oral keratinocyte cultures to periodontal pathogens elicited EMT-like properties, which indicates that this process may promote loss of epithelial integrity (250).

## Conclusions

The composition of the gingival epithelial barrier is quite complex since its structure composes of a huge number of different molecules. Keratins are the major component of the keratinizing stratified epithelial cytoskeleton. Depending on localization and function different epithelia express a distinct cytokeratin pattern. In order to sustain their function, stratified epithelia, including the oral mucosa, have to sustain tight cell-cell adhesion in the viable cells that involves intercellular tight and adherens junctions which connect to the actin cytoskeleton. Oral tissue immune response is able to recognize microbial infection and colonization and is able to manage it. The epithelial cells express a number of pattern recognition receptors, including TLRs, NOD1, NOD2 and PARs and are able to assemble different kinds of inflammasomes and express a variety of pro-inflammatory cytokines and chemokines. Resident oral bacteria permanently influence epithelial host cells. Depending on their composition biofilms differentially modify cellular immune responses of the epithelium. Major periodontal pathogens like *P. gingivalis* possess a number of different strategies to escape from innate immunity and survive in the tissues, which affects the epithelial barrier by modifying the expression and integrity of the different cell-cell junctions.

The balanced immune-inflammatory state of the host with its biofilm in health may be disturbed by distinct species, such as *P. gingivalis* and *F. nucleatum* that are able to destroy this equilibrium, causing a dysbiotic microbiota (251). Chronic infections and persistent inflammation are associated with an increased risk of cancer. Persisting bacterial agents may induce up-regulation of immune-inhibitory receptors which in turn facilitate the ability of cancer cells to evade from anti-tumor immune responses of the host. Furthermore, long term infection possibly supports carcinogenesis by regulating gene expressions of the infected epithelial cells in a way that leads to development into a phenotype indicating cellular transformation from normal to cancerous.

## Author Contributions

SG wrote the manuscript. JM corrected the manuscript and assisted in writing.

### Conflict of Interest Statement

The authors declare that the research was conducted in the absence of any commercial or financial relationships that could be construed as a potential conflict of interest.
